# Cancer Burden in Adolescents and Young Adults in Belgium: Trends to Incidence Stabilisation in Recent Years with Improved Survival

**DOI:** 10.3390/cancers17091543

**Published:** 2025-05-01

**Authors:** Fabienne Van Aelst, Bart Van Gool, Nancy Van Damme, Hélène A. Poirel

**Affiliations:** 1 Cancer Center, Sciensano, 1050 Brussels, Belgium; helene.antoine-poirel@sciensano.be; 2 Belgian Cancer Registry, 1210 Brussels, Belgium; bart.vangool@kankerregister.org (B.V.G.); nancy.vandamme@kankerregister.org (N.V.D.)

**Keywords:** adolescents and young adults, AYA, incidence, survival, prevalence, mortality, cancer epidemiology, cancer trends, Belgium

## Abstract

**Background/Objectives**: This population-based study examined epidemiological trends of primary cancers in adolescents and young adults (AYAs) to enhance the understanding of the specific spectrum of cancers impacting AYAs in Belgium. **Methods**: Data on incidence, prevalence, mortality, and survival were obtained from the Belgian Cancer Registry (2004–2020, N = 43,535). (A)APC statistics were compared with children (5–14 years) and adults (40–49 years). **Results**: Cancer incidence increased by 0.4% annually from 66 to 80 per 100,000 person-years (ESR2013) but stabilised after 2015, except for Hodgkin lymphoma, chronic myeloid neoplasms, and testicular and breast cancer, which continued to rise. Mortality decreased by 1% annually, from 10 to 7 per 100,000 person-years (2004–2019). Five-year relative survival (RS) was 87% but remained low for certain cancers, including ovary (78%), central nervous system (67%), precursor haematopoietic neoplasms (64%), gastrointestinal (excl. colorectal) (49%), and lung-bronchus-trachea cancers (42%). **Conclusions**: From 2004–2020, the cancer burden among AYAs in Belgium increased due to improved survival, while incidence stabilised after 2015. Five-year RS exceeds 80% overall but remains lower for some cancers compared to children (e.g., precursor haematopoietic neoplasms) or older adults (e.g., breast cancer, sarcoma). The Belgian epidemiological trends align with those in neighbouring countries (Netherlands, France, Germany).

## Simple Summary

While the cancer burden in adolescents and young adults (AYA) is increasing, cancer is the fourth leading cause of death among AYA globally. AYA represent a distinct patient population that is often overlooked in favour of paediatric and older adult patients. It is therefore crucial to better understand AYA cancers. This study aims to provide an overview of the specific epidemiological trends of AYA (15–39 years) cancer patients in Belgium over the period 2004–2020 by age sub-groups and by placing these trends in an international context. The increased cancer burden is mostly due to improved survival, while incidence is stabilised after 2015. Although varying by cancer type, survival outcomes for AYAs are generally lower than those observed in children with similar malignancies and not systematically better than in the older age group, 40–49 years. These results offer valuable insights to support policymakers in optimizing AYA cancer management in Belgium.

## 1. Introduction

In Belgium, cancer predominantly affects older individuals, with 70% of women and 81% of men aged 60 years or older at diagnosis in 2022 [1]. Given that approximately 19% of the Belgian population is 65+, these figures highlight the disproportionately high cancer incidence among older adults [2]. Although cancers in adolescents and young adults (AYAs) are rare, they represent the fourth leading disease-related cause of death in this population in high- and middle-income countries [3]. Trama et al. (2023) [4] reported approximately 112,000 new cancer cases among AYAs in Europe, representing 5% of the new cancers diagnosed in the total population of the EU countries selected for their study. Gupta et al. (2020) [5] studied incidence trends of AYA cancers in 41 countries, spread across the continents, for the period 1998–2012. Data from the International Agency for Research on Cancer’s CI5plus database [6] were used. The authors reported an increase in AYA cancer incidence in 23 countries, predominantly industrialised ones, with colorectal, thyroid, testicular cancer, and melanoma types (particularly in European countries) being the highest contributors. You et al. [7] used data from the Global Burden of Disease 2019 study [8] to assess epidemiological trends for AYA cancer in 204 countries, from 1990 to 2019. The authors stated that the incidence of cancer in AYAs increased slightly by 0.4% annually in Western Europe.

EUROCARE-5 demonstrated that survival rates in AYAs trail behind those in children for several cancers that affect both groups, especially for more common haematological malignancies [9]. Inferior survival rates of AYAs have been attributed to various patient- and system-level factors, such as individual behaviours (e.g., lower treatment adherence), differences in biological characteristics or pathophysiology, variations in the pharmacokinetics of chemotherapeutic agents, less inclusion of AYAs in clinical trials, and delays in diagnosis and treatment [10,11]. Additionally, other important attributing factors are the lack of specialised guidelines and education and inadequate tailored healthcare professional attention and awareness [12].

The definition of AYAs with cancer is not fixed and varies deeply according to countries. The age range of adolescence (15–19 years) is generally accepted in the literature, even though it is sometimes suggested to start younger, from 14, 13, or puberty. There is, however, still a lack of consensus concerning the upper age limit of young adulthood, varying from 20 to 39. In 2006, the United States National Cancer Institute’s (NCI) Adolescent and Young Adult Oncology Progress Review Group defined AYAs with cancer as those diagnosed at age 15–39 years [13]. This definition was adopted by the European Network for Cancer in Children and Adolescents (ENCCA) and the Joint Cancer in the AYA ESMO/SIOPE Working Group. Heterogeneous age groups are currently used in different European countries. The AYA definition influences the way this age group is managed, depending on the national/regional cancer care organisation, either in the adult ward or in the paediatric setting, or a specific AYA unit [14]. In Belgium, the AYA age group has been defined as 16–35 years, per the RIZIV-INAMI convention on psychosocial care for AYAs [15], where 16 corresponds to the upper age limit for paediatric wards.

Cancers in AYAs are distinguished from those in children and older adults because of important differences in the distribution of cancer types, risk factor profiles, cancer biology, survivorship, and long-term health consequences [10]. AYA cancers represent a heterogeneous spectrum of malignancies, with the distribution of cancer types varying significantly by age, ranging from paediatric cancers like acute lymphoblastic leukaemia (ALL) to adult cancers such as carcinoma and melanoma. Moreover, some cancer types exhibit an incidence peak in the AYA age group, such as testicular cancers and Hodgkin lymphoma [10,16]. Due to this heterogeneity in cancer types, overlapping with children and older adult cancer types, AYA patients fall between the paediatric and adult oncology or haematology care settings. As a result, this group is generally overlooked and understudied [12].

Classification of cancers diagnosed in adulthood mostly relies on locations, while in paediatrics, cancers are grouped by main morphology types [17]. Since AYA cancers are specific and intermediate between paediatric and older adult cancers, the Surveillance, Epidemiology, and End Results Program (SEER) proposed another way to group the ICD-O codes (combination of diagnostic and morphology criteria) to fit the specific distribution of cancer types in AYA. The SEER AYA site recode classification scheme is adapted from the classification scheme proposed by Barr et al. [16,18].

The distinction between AYA and other age categories is not restricted to diagnosis and survival but also concerns the psychosocial level. AYAs find themselves in a very specific stage of life with a life-threatening disease and therefore experience specific needs and problems. Young cancer patients and survivors often face long-lasting physical side effects and psychological consequences. These influence quality of life, social life, professional/student relationships, and intimate relationships negatively [19,20].

The Belgian Cancer Registry (BCR) is a national and population-based cancer registry that has collected and maintained epidemiological data on cancer across the country since 2004. BCR regularly releases reports on cancer incidence, prevalence, and survival in Belgium on the BCR website. In addition to three reports released in 2013, 2019 and 2023 that focused on cancers diagnosed in children and adolescents (0–19 years old) [21,22,23], a recent report [24] presents epidemiological data on cancers in AYAs, with the age range defined by the previously mentioned RIZIV-INAMI Convention [15] aged between 16 and 35 years.

This study is the first to provide a broad perspective on the epidemiological trends of AYA cancer patients in Belgium over time, by age sub-groups, and by placing these trends in an international context. Using the international AYA definition (15–39 years) and standardised cancer classifications, meaningful international comparisons were facilitated.

## 2. Materials and Methods

### 2.1. Data Source and Selection

Cancer registration in Belgium has a legal basis. In 2006, the law concerning the Cancer Registry was created, making cancer registration compulsory for oncological care programs and laboratories for pathological anatomy [25,26]. Through linkage with the Crossroads Bank for Social Security, the Cancer Registry can actively follow up on the vital status and date of death of the patients.

All malignant neoplasms (ICD-O-3 behaviour code/3) and all neoplasms, whatever the behaviour (/0 benign, /1 intermediate), for CNS tumours in Belgian residents, aged 15–39 years and diagnosed between 2004 and 2020, were extracted from the BCR database. Incidence data with ICD-O-3 classification were grouped according to the SEER AYA site recode classification scheme [18]. The classification is filed in Table S1. Cancers classified under the SEER code 10 (‘Miscellaneous specified neoplasms’) and 11 (‘Unspecified malignant neoplasms except for CNS’) were grouped and renamed to ‘Other Neoplasms’.

For the generation of mortality data (which are classified according to the ICD-10 classification), the grouping was adapted to match as much as possible the ICD-O-3-derived SEER classification. Haematological malignancies could not be subdivided due to a discrepant subclassification by diagnosis, location, and topography. Belgian population data are obtained from the Directorate-General for Statistics Belgium (Statbel) [27]. Data transmitted by Statbel, available up to the year of incidence 2019, were used to generate mortality data. ICD-10 is a classification for all diseases, not only cancers. Statbel provides data exclusively on malignant neoplasms (group C) in an aggregated format, with BCR utilised to estimate cancer mortality in Belgium. Sex, age, and calendar year-specific population data were retrieved from Statbel as well.

Although the AYA age group is defined as 16–35 in Belgium, the age range 15–39 years—of the US NCI [13] and the Joint Cancer in AYA ESMO/SIOPE WG [28]—was selected for this study, as this definition is widely used in international literature and consequently enables making a comparison between Belgium and other countries. To overcome analyses with small numbers, the data were aggregated in age groups (15–19 years, 20–24 years, 25–29 years, 30–34 years, 35–39 years) for the calculation of Belgian incidence, prevalence, mortality, and survival data. The rates for each sex were presented separately whenever possible. Otherwise, data for men and women were grouped. Results of children (5–14 years) and older adults (40–49 years) were also included to allow comparison of AYA trends and specificities with their closest age groups. Younger children, especially infants (<5 years old), and elderly adults (>50 years old) have very different characteristics and prognostic factors which may modify survival and incidence.

Carcinomas of the gastrointestinal (GI) tract, except colorectal and pancreas carcinomas, (SEER code 9.3, carcinomas of the oesophagus, stomach, small intestine, liver-intrahepatic bile ducts, pancreas) were kept combined under the term ‘Rest of the carcinomas of GI tract’ and were seen in small numbers in the AYA population.

To enable international comparisons of trends in incidence and mortality, the (A)APC values were calculated for France, Germany, and the Netherlands. The data sources for these countries were as follows: (1) the Netherlands: population data were obtained from the Centraal Bureau voor Statistiek [29], while mortality and incidence data came from the Integraal Kankercentrum Nederland [30]; (2) France: population data were sourced from the Institut National de la Statistique et des Etudes Economiques [31], and mortality and incidence data were obtained from the Institut National du Cancer [32]; and (3) Germany: population data were retrieved from DeStatis Statistisches Bundesamt [33] and mortality and incidence data were obtained from Zentrum Für Krebsregisterdaten [34].

### 2.2. Analysis

Descriptive analyses were performed on the data provided by BCR: the total cases for incidence, mortality, 5- and 10-year prevalence, and 5-year relative survival. Annual-specific rates were calculated per 100,000 person-years by BCR using the mid-year population size obtained from Statbel. The total studied period is 17 years (2004–2020). Incidence, mortality, and prevalence rates were age-standardised (ASR) with weights based on the five-year age groups from the 1976 and 2013 European standard population (ESP) data [35] and the world standard population (WSP) data [36]. Since the structure of the Belgian AYA population is comparable to the ESP 2013, the ESR 2013 was used as the ASR throughout this study. For sections comparing the results internationally, the WSR was used. In the Supplementary Materials, the results are shown in crude rates, ESR, ESR2013, and WSR.

Relative survival data were calculated and provided by BCR. One-, five- and ten-year relative survival rates by sex and age group (15–19, 20–24, 25–29, 30–34, 35–39 years old) are presented and compared with a younger age group (5–14 years) and an older age group (40–49 years). Expected survival calculations were based on sex, age, and calendar-year-specific Belgian life tables [27], according to the Ederer II method. This method is based on the actual follow-up time rather than the potential follow-up time to estimate survival [37]. Relative survival data with N at risk <50 were not included in the analysis because they are below the statistical reliability threshold.

For the international comparison, annual-specific rates were calculated per 100,000 person-years by direct standardisation. Incidence and mortality rates were age-standardised (ASR) with weights based on the five-year age groups from the 2013 European standard population (ESP2013).

Since data have been collected from 2004 onwards, results can also be compared over time. In total, 17 consecutive years of incidence and 16 years of mortality data were used in this study. Because of the low incidence of cancers in the AYA population, incidence and mortality rates are aggregated over two age groups, 15–29 years and 30–39 years. To assess the average annual percentage change (AAPC), the freely available Joinpoint Regression Program version 5.0.2 [38] was used by fitting joinpoint models to the log-transformed age-standardised incidence and mortality rates. All models were plotted using the ‘grid search method’ and the ‘uncorrelated error model’. The number of points to place between adjacent observed x-values in the grid search method was set at 2 (the default is 0). The uncorrelated error model was selected, as correcting for autocorrelation can reduce the power to detect joinpoints. A minimum of zero and a maximum of two joinpoints were allowed in this 16- to 17-year analysis [39]. Selection of the final model was done using the recommended Bayesian information criteria 3 (BIC3). Further parameters within the Joinpoint program were kept at their default setting.

## 3. Results

### 3.1. Incidence

A total of 43,535 patients aged 15–39 years were diagnosed with cancer (all cancers excluding non-melanoma skin cancer) in the period 2004–2020 in Belgium. Overall, AYAs represented 4% of the Belgian cancers diagnosed in the studied period. The age distribution in this cohort was 6%, 10%, 17%, 26%, and 41% in the respective age groups 15–19, 20–24, 25–29, 30–34 years, and 35–39 years old at diagnosis (Table S2).

The overall age-adjusted incidence rates were higher in female AYAs than in male AYAs, with a female-to-male ratio of 1.65 (94 vs. 57 cases per 100,000 population, during 2004–2020). Cancer incidence rates are slightly higher in male AYAs aged 15 to 19 years (25 vs. 22 cases per 100,000 population in males and females, respectively, during 2004–2020), mostly because of testis cancer, sarcoma, and haematological neoplasms (4, 4, and 3 cases per 100,000 population, respectively). The rates did not differ between the sexes in the 20–24 age group (38 cases per 100,000 population for males and 39 for females). Rates in women compared with men, however, are 30% higher in AYA aged 25 to 29 years (69 vs. 54 per 100,000 population) and nearly double in those aged 30 to 39 years (158 vs. 79 per 100,000 population), primarily because of the substantially higher incidence of breast, thyroid, and genital cancers (mostly cervix cancer), and melanoma of the skin in women. For example, thyroid cancer incidence rates among women in their late twenties are almost fivefold higher than those among men (nine vs. two per 100,000 population during 2004–2020, respectively).

Among AYAs, incidence increases with advancing age for all cancer types except for Hodgkin lymphoma, precursor haematopoietic neoplasms, testis tumours, and histiocytic and dendritic cell neoplasms (HDCN).

**Figure 1:**
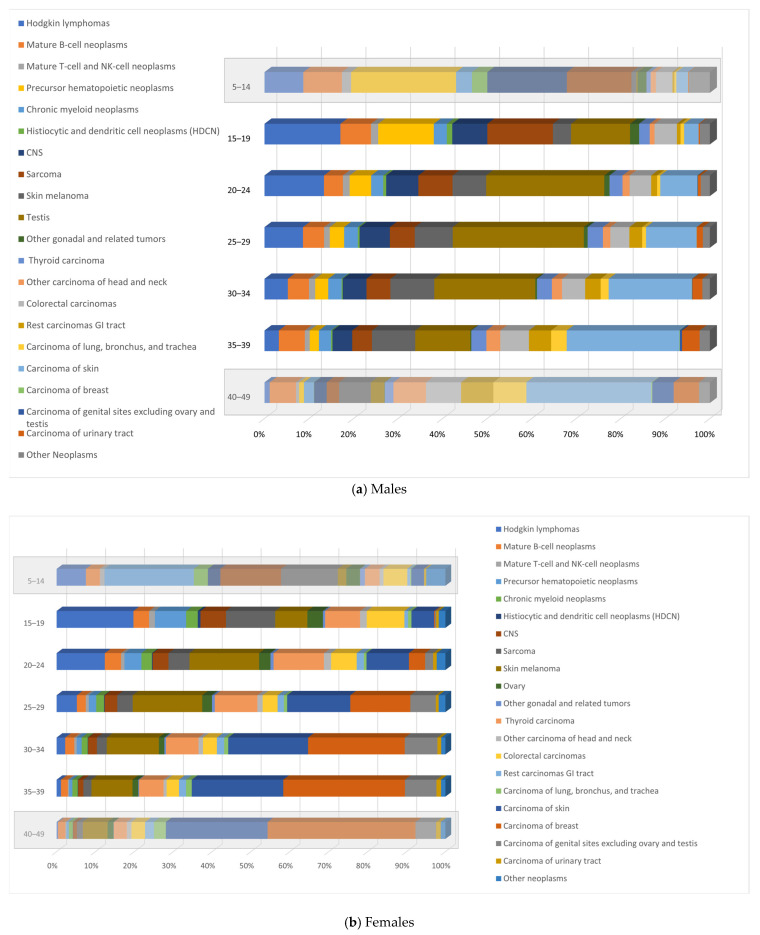
Relative frequencies (%) of cancer types by age group for (**a**) male and (**b**) female cancer patients, Belgium, 2004–2020.

In general, the cancer type distribution shifted considerably with age (Figure 1) from a higher proportion of childhood cancers (e.g., precursor haematopoietic neoplasms, mostly acute lymphoblastic leukaemia/lymphoma—ALL/L) in adolescents (individuals aged 15–19 years) to more adulthood cancers in the 35–39 young adults (e.g., carcinomas). Moreover, some cancer types occur more specifically in the AYA subgroup: Hodgkin lymphoma and testis tumours exhibit a peak of incidence in AYAs, in the age groups 20–24 years old and 25–34 years old, respectively.

### 3.2. Mortality

**Figure 2:**
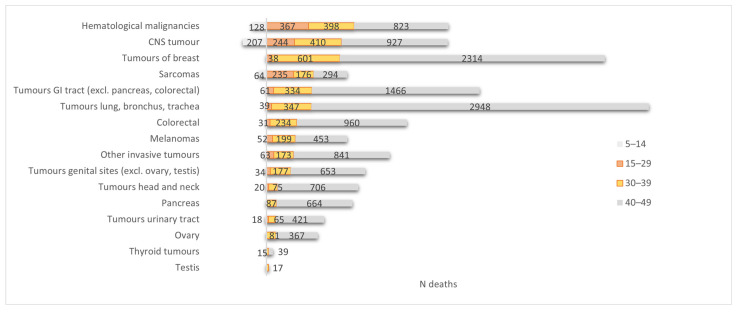
Cancer causes of death in children (5–14 years), AYAs (15–39 years), and older adults (40–49 years), Belgium, 2004–2019.

A shift with age is also observed for mortality by cancer type. During the period of 2004–2019, haematological malignancies, CNS tumours, and sarcoma were among the leading cancer causes of death in younger AYAs (aged 15–29 years), similar to those in children (5–14 years). In the older AYAs (aged 30–39 years), the top five leading cancer causes of death were similar to those identified in the older adults (40–49 years) but not in the same order: breast cancer was followed by CNS tumours and haematological malignancies, while cancer of the lung, bronchus, and trachea was the leading cancer cause of death, followed by breast cancer and CNS tumours (Figure 2 and Table S3).

### 3.3. Long-Term Trends in Incidence and Mortality

**Figure 3:**
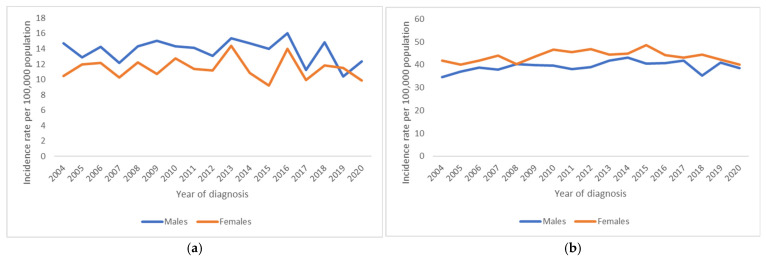

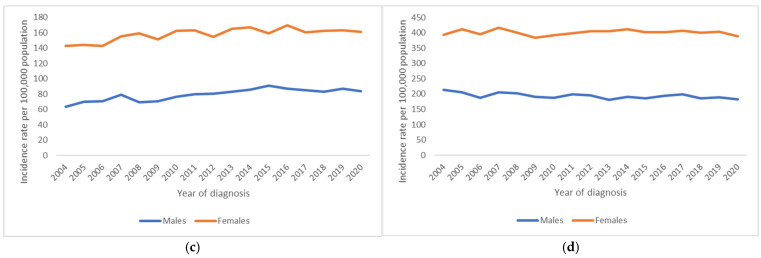
Trends in cancer incidence rates for all cancers combined, 2004 to 2020, Belgium. Rates are age-adjusted to the 2013 European standard population. (**a**) Children aged 5–14 years, (**b**) AYAs aged 15–29 years, (**c**) AYAs aged 30–39 years, (**d**) adults aged 40–49 years.

Long-term trends in cancer incidence and mortality rates by age group among AYAs compared to children and patients in their forties are shown in Figure 3. The age-standardised incidence rates among AYAs slightly increased by 0.2% per year from 2004 to 2020 (Table S4). However, breaking down the trend, it is observed that this increase occurs from 2004 to 2015, as from that year on, the rates become too scattered, and a trend could not be detected. In younger women (15–29 years old), the incidence has even moderately decreased by −1% per year since 2015. It is noteworthy that the incidence remained stable over the whole studied period for patients in their forties, while no significant trend could be observed over time for children, mainly due to a lack of robustness.

Four cancer types showed an increase in incidence during 2004–2020. The moderate increase in incidence for breast cancer in AYAs (0.3% per year) during the whole period of 2004–2020 seems largely attributable to the rising incidence rates in the younger AYAs (15–29 years, 2% per year). At the same time, the incidence in older adults (40–49 years) slightly declined (−0.1% per year). Testis cancer increased over time for AYAs (1% per year), as well as for patients in their forties (3% per year). A significant rising trend was detected for the incidence rates of Hodgkin lymphoma in AYAs (+1% per year) over the 17 years. In older adults (40–49 years), no significant trend could be observed over time for this cancer type. The incidence of chronic myeloid neoplasms increased over time for AYAs (+3% per year), as well as in patients aged 40–49 years (+1% per year). The incidence decreased over time for carcinoma of genital sites, excluding the ovaries and testes (mostly cervical cancer), by −1% per year.

For 3 cancer types, the incidence initially increased, but from a certain point in time, either the incidence rates became too scattered and a significant trend could not be observed, or the incidence rates decreased significantly. This was the case for thyroid cancer (+4% per year until 2012, from then on a decrease of 1% per year), colorectal cancer (increase of 3% per year, and from 2013 no trend), and skin melanoma (+2% per year and from 2010 onwards no trend).

**Figure 4:**
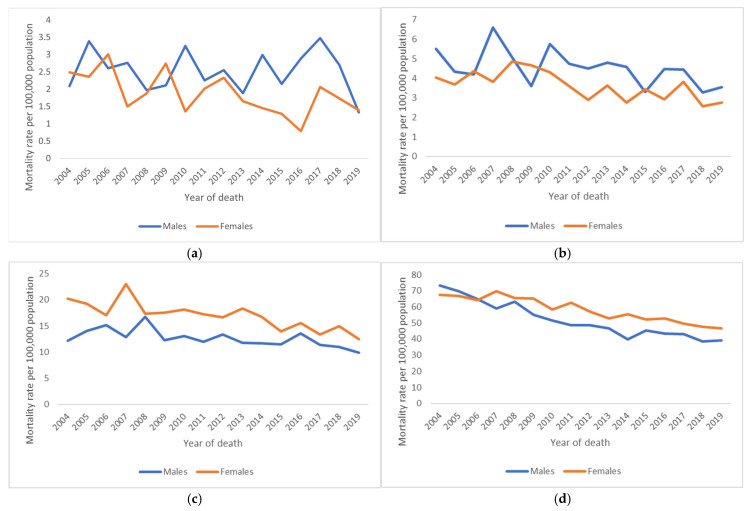
Trends in cancer mortality rates for all cancers combined, 2004 to 2019, Belgium. Rates are age-adjusted to the 2013 European standard population. (**a**) Children aged 5–14 years, (**b**) AYAs aged 15–29 years, (**c**) AYAs aged 30–39 years, (**d**) older adults aged 40–49 years.

However, a trend in incidence could not be observed in the studied 17-year period for most cancer types, e.g., CNS tumour, sarcoma, non-colorectal cancer of the GI tract, lung/bronchus/trachea cancer, urinary tract carcinoma, head and neck cancer, gonadal non-testicular tumours (mostly ovarian cancer), and precursor haematopoietic and mature B-cell neoplasms. The Belgian mortality rates in AYAs decreased significantly by 1% per year (Figure 4 and Table S4). Whereas a trend in mortality over time in children could not be detected, the mortality rates decreased in older adults by 1% per year.

The Belgian mortality rates decreased significantly for all AYA age groups and both sexes in the studied period 2004–2019, where cancer mortality rates were generally higher for female AYAs. The mortality decreased more in the younger AYA group (15–29 years, −2% per year) compared to the older AYA group (30–39 years, −1% per year). Due to low numbers, mortality trends for most cancer types could not be calculated. Nonetheless, a decrease in mortality since 2004 was observed in older AYAs (aged 30–39 years) with breast cancer (−3% per year), where the mortality decreased faster than in patients aged 40–49 years (−2% per year). Significant rising trends could not be observed.

### 3.4. Relative Survival

**Figure 5:**
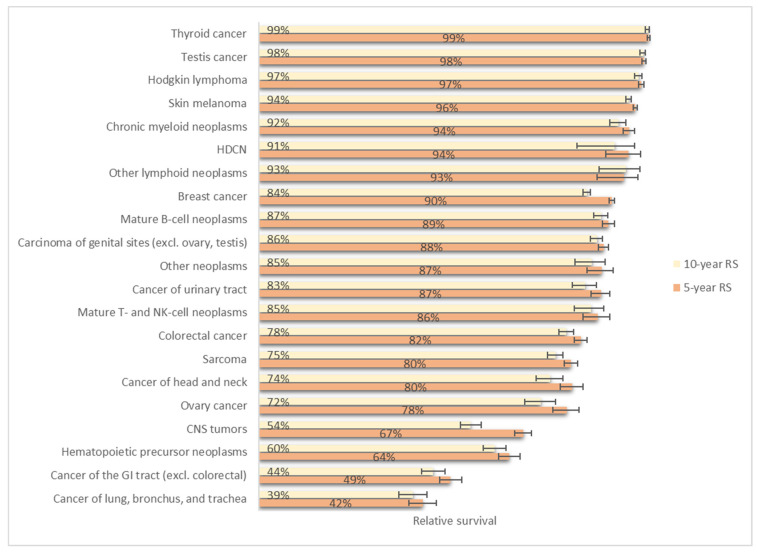
Five- and ten-year relative survival (with 95% CI) per cancer type, Belgium, 2004–2020.

The short-term 1-year relative survival (RS) rates for all cancers together, diagnosed between 2004 and 2020, show a very good result, with 94% one-year RS in males and 97% in females, aged 15–39 years (Table S5). The slight difference in survival for males versus females is confirmed in the longer term; the overall five-year male-female RS (15–39 years) is 84% versus 89%, and it is 81% versus 85% for the 10-year RS. The long-term RS rates for females remain above 80% for all age groups, while they drop below 80% in males from age 35 (79% and 75% at 5- and 10-year RS, respectively). Although the RS rates (1-, 5- and 10-year RS) remained above 90% for all age groups for Hodgkin lymphoma, chronic myeloid neoplasms, melanoma, testis cancer, HDCN, thyroid carcinoma (Figure 5), 5-year RS remained under 80% for AYAs with ovarian cancer (78%), CNS (67%), precursor haematopoietic neoplasms (64%), non-colorectal carcinoma of the GI tract (49%), and carcinoma of the lung-trachea-bronchus (42%).

Whereas the 5-year RS for most cancer types decreased with age (haematological malignancies, lung/bronchus/trachea, ovarian cancer, cancer of the GI tract, testis cancer, CNS tumours) or remained stable over the age groups (thyroid carcinoma, testis cancer, skin melanoma), it slightly increased with age for sarcomas and breast cancer, with the poorest survival outcome in adolescents (sarcoma) and 20–24-year-olds (breast cancer), compared to older adults (5-year RS of respectively 70% vs. 82% in sarcoma and 87% vs. 94% in breast cancer). The 5-year relative survival (RS) is higher in AYAs than in children and older adults for chronic myeloid neoplasms (94% in AYAs, 88% in children, 92% in older adults) and CNS tumours (67% in AYAs, 63% in children, 43% in older adults).

### 3.5. Prevalence

It was estimated that on 31 December 2020, 9722 AYAs (3929 males, 5793 females) who had been diagnosed with at least one type of cancer (excluding non-melanoma skin cancer) within the past 5 years were living in Belgium, and there were 15,192 (6358 males, 8834 females) who had been diagnosed within the past 10 years. This represents, respectively, 0.3% and 0.4% of the Belgian AYA population in 2020. Prevalence data are detailed in Table S6.

**Figure 6:**
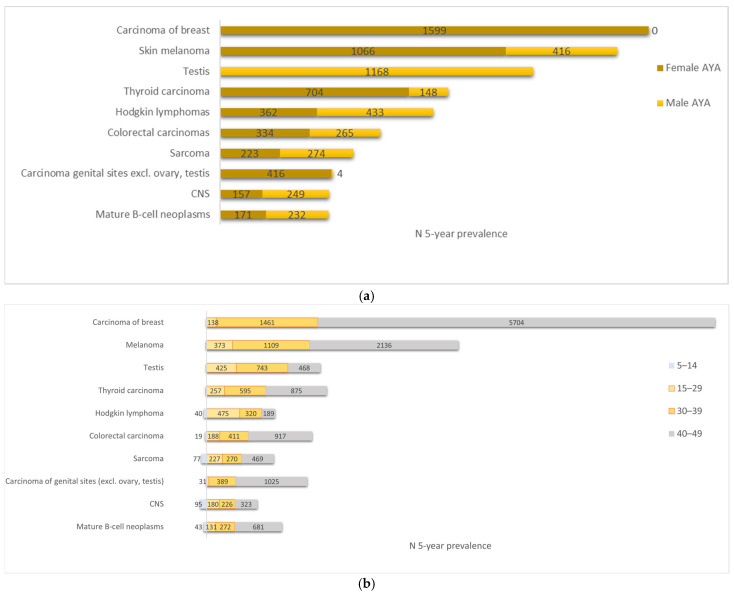
The ten most prevalent 5-year cancer types (excl. carcinoma of skin) in AYAs: (**a**) per sex, AYAs 15–39 years; (**b**) both sexes, per age group (children (5–14 years), AYAs (15–29 years and 30–39 years), older adults (40–49 years)), Belgium, 31 December 2020.

The five primary cancers with the highest 5-year prevalence in Belgian AYAs (15–39 years) were carcinoma of the breast, skin melanoma, testis cancer, thyroid carcinoma, and Hodgkin lymphoma (Figure 6). The distribution of the most prevalent cancers also shifted with age. The youngest age group (15–29 years) was composed of a mix of cancers highly prevalent in children, as well as other cancers, such as melanoma, testis, and thyroid cancers. These three latter cancer types stay in the top five most prevalent cancers in AYAs older than 20 years. The most prevalent cancer in these younger age groups was Hodgkin lymphoma. Sarcoma was the second most prevalent cancer in adolescents (15–19 years), followed by precursor haematopoietic neoplasms, CNS, and mature B-cell neoplasms. From age 25 onwards, breast cancer introduces itself and is the most prevalent cancer in AYAs older than 30. Cancer of genital sites (excl. ovary, testis)—mostly cervical cancer—is highly prevalent in AYAs older than 30 years. Colorectal cancer can be found in the top ten most prevalent cancers in children, AYAs, and older adults.

### 3.6. International Comparison

**Figure 7:**
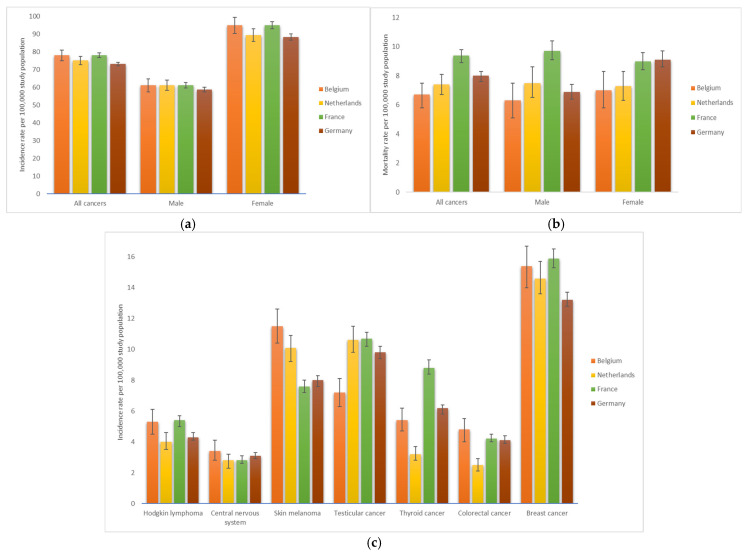
Comparison of Belgian incidence and mortality rates in 2019 with those of the Netherlands, France, and Germany. Rates are age-adjusted to the 2013 European standard population. (**a**) Incidence of all cancers, (**b**) mortality from all cancers, and (**c**) incidence of selected cancer types.

When comparing the Belgian incidence and mortality data with the neighbouring countries (the Netherlands, France, and Germany), the rates in 2019 are aligned (Figure 7, Tables S7 and S8); no statistical differences were detected when comparing the Belgian rates with those of the three other countries. The year 2019 (instead of 2020) was selected for comparison to avoid any potential impact of the COVID-19 pandemic on the interpretation of the results. While Belgian incidence rates are comparable to those in the Netherlands, France, and Germany, overall cancer mortality rates in Belgium are relatively lower than in neighbouring countries. Mortality rates in AYAs have decreased in Belgium as well as in surrounding countries and at a somehow identical percentage in Belgium, the Netherlands, and France (−1% per year) (Table S7).

The incidence trends are generally aligned (Table S7). While no clear trend in Hodgkin lymphoma incidence was observed in the Netherlands, an increase of approximately 1% per year was noted in Belgium, France, and Germany. Over the last decade of the observed period, incidence rates for skin melanoma remained relatively stable in Belgium but showed a significant decline in the Netherlands, France, and Germany, with annual decreases of −1%, −1%, and −2%, respectively. In 2019, the incidence of skin melanoma in Belgium (12 per 100,000 population) was slightly higher than in the Netherlands (10 per 100,000) and France and Germany (both 8 per 100,000 population). While colorectal cancer incidence remained relatively stable in Belgium over the last decade of the observed period and in the Netherlands from 2004 to 2020, it showed a gradual increase in Germany and France, with annual rises of +1% and +3%, respectively. In 2019, Belgium had a lower incidence of testicular cancer (7 per 100,000) compared to the Netherlands, France (11 per 100,000), and Germany (10 per 100,000). Belgium’s incidence increased by 1% annually, similar to France (+2% per year). In the Netherlands, the trend initially rose but stabilised from 2014 to 2020, while no trend was observed in Germany. Comparable incidence trends for thyroid cancer were observed in Belgium, the Netherlands, France, and Germany. Initially rising, the incidence then either decreased (Belgium since 2012 and France since 2013) or stabilised (Netherlands since 2014 and Germany since 2009). Between 2004 and 2020, breast cancer incidence in Belgium increased by 0.3%, a trend also observed in France (+0.5%), the Netherlands, and Germany (both + 0.3%).

## 4. Discussion

This study provides insight into the epidemiological trends among AYAs aged 15 to 39 years at diagnosis of cancer in Belgium, between 2004 and 2020. The key findings are as follows:

(I) The spectrum of cancers in AYAs is greatly distinct and heterogeneous, justifying the importance of distinguishing AYAs based on age and sex instead of grouping them all together for analytical purposes.
(II) The standardised incidence trend has stabilised in the more recent years after a slightly increasing trend between 2004 and 2015, with variations depending on cancer types as well as on sex and age.
(III) The Belgian incidence and mortality trends align with those observed in surrounding countries, while the mortality rate for AYAs in Belgium generally remained lower than in neighbouring nations.

The results of this study are in line with Trama et al. [4], reporting that testis cancer is the most common cancer among young men aged 15–39 years, and Li et al. [40], reporting that among women, early-onset breast cancer has the highest incidence. The Belgian incidence rate for skin melanoma was slightly higher than the European average reported by Trama et al. but is comparable to the neighbouring countries of Germany and the Netherlands [4]. The higher cancer incidence rate in female AYAs is mostly explained by the higher incidence of thyroid and breast cancers in female AYAs in the 30–39 age subgroup. The relatively higher cancer incidence rates among female AYAs older than 20 years, compared to male AYAs, have been observed in Europe and globally [4,40].

The **incidence** rates in the Belgian AYA population generally rose from 2004 to 2015. This finding aligns with Trama et al. [4], who reported a steady rise in European incidence (1998–2012).

Although the aetiology of early-onset cancer is still uncertain, it can most likely be linked to exposure to risk factors at an early age. Risk factors are multifactorial, resulting from a combination of environmental and lifestyle factors in addition to a genetic susceptibility, with differences between the specific cancer types, sex, and age [41,42]. The genetic predisposition of cancer in AYAs (as well as in childhood) is higher than in older patients [43]. The contribution of established risk factors to the increasing incidence of AYA cancer is uncertain, necessitating additional research. It might be that a better survival of childhood cancers and the higher risk of developing subsequent malignancies in survivors when compared with the general population [44] might slightly contribute to this increase in AYA cancer. Macq et al. [45] studied the epidemiology of multiple primary cancers in Belgium and found that the odds of having more primary cancers increase with age and depend on the cancer site. However, this study could not test this hypothesis.

Since 2015, however, the Belgian incidence trend has stabilised and even decreased in female AYAs aged 15–29 years. This observation aligns with Zhang et al. [46], who analysed epidemiological trends among US AYAs from 2016 to 2021 and found that after a period of rising incidence rates, they stabilised from 2016 onward. Additionally, Voeltz et al. [47] observed similar incidence trends, reporting a rise in German early-onset cancer cases from 1990 to 2010, followed by a decline from 2010 onward.

This plateauing trend in Belgian incidence is mainly driven by the stabilisation or decline in incidence rates for skin melanoma, thyroid cancer, and colorectal cancer, starting from 2010, 2012, and 2013, respectively.

The incidence rates for **skin melanoma** in Belgian AYAs are relatively high, compared to the European rate [4]. Where the incidence steadily increased over time for AYAs aged 30–39 with 1% per year, it declined from 2010 onward in younger AYAs (aged 15–29). Similarly, Zhang et al. [46] found that from 2016 onward, US incidence for this cancer type decreased in AYAs. The prominent increases in skin melanoma among older AYAs (aged 30–39) over time likely result from (unprotected) increased sun exposure. It could also reflect increased cancer awareness, resulting in more detection as well as more systematic registration. Earlier studies suggest that younger people might be more biologically susceptible to the carcinogenic effects of artificial UV radiation, increasing the risk of early-onset melanoma in a dose-response way with first exposure at an early age and frequency of exposure [48]. The decrease in incidence in younger AYAs (15–29 years old) since 2010, on the other hand, might indicate that primary preventive actions in early life (e.g., education on sun protective behaviour) are effective [49]. Despite this positive message, the Belgian organisation Foundation Against Cancer warns that awareness amongst 16–24 year olds has been decreasing in recent years [50].

It remains unclear what exactly causes the higher **thyroid cancer** incidence among female AYAs [51]. The incidence rose significantly by 4% per year until 2012, after which it declined by 1% annually. In older patients, aged 40–49 years, the initial change in incidence stabilised from 2015 onward. A stable trend was observed in the US for this cancer type in AYAs between 2016 and 2021 [46]. However, in the Netherlands [52] (AYA, 1990–2016) and in Germany [47] (early-onset, 1999–2019), an upward trend was observed in incidence for this cancer type. There is some evidence suggesting that exposure to ionising radiation in childhood could influence the incidence of thyroid carcinomas in AYAs [53]. However, in low-risk regions, such as Belgium, increasing incidence trends in thyroid carcinoma could be attributed to overdiagnosis [54]. Van den Bruel et al. [55] showed in 2013 that regional variations in the incidence of thyroid cancer in Belgium were attributable to variations in imaging detection rates, the latter leading to overdiagnosis.

Belgian incidence rates of **colorectal cancer** in AYAs increased by 3% per year until 2013, stabilising from that year on. The incidence of this cancer type remained somewhat stable in Belgian adults aged 40–49 years. The 5-year RS for the overall group of AYAs remains above 80%; however, there is a considerable difference in survival between AYAs aged 15–29 years (91%) and those aged 30–39 years (77%). Delays in the detection of cancer for this age group might lead to more progressed stages of cancer at the time of the diagnosis [56]. Dutch incidence rates for CRC in AYAs increased between 1990 and 2016 [52]. Voeltz et al. [47] observed, on the other hand, stable incidence trends in early-onset colorectal cancer (Germany, 1999–2019). Zhang et al. [46] reported an increase in rectal cancer and a stable trend in incidence of colon cancer in AYAs over 2016–2021. An increase in colorectal cancer among young European adults from 1990 to 2016 was reported by Vuik et al. [57]. Modifiable risk factors, such as a Westernized diet (processed meat, low vegetable, fish, and fruit intake), obesity, alcohol intake, smoking, and lack of physical activity, were found to be associated with early-onset colorectal cancer, as well as non-modifiable factors such as male sex, Caucasian race, inherited predisposition, and personal history of inflammatory bowel disease [58,59]. Early-onset colorectal cancer has a unique biology relative to late-onset colorectal cancer, exhibiting more aggressive histological subtypes, such as mucinous and signet ring cell carcinomas [60].

Most cancer types showed stable trends in incidence from 2004 to 2020. This is the case for CNS tumours, sarcoma, non-colorectal cancer of the GI tract, lung/bronchus/trachea cancer, urinary tract carcinoma, head and neck cancer, gonadal non-testicular tumours (mostly ovarian cancer), and precursor haematopoietic and mature B-cell neoplasms.

From 2004 to 2020, incidence rates for **CNS tumours** remained stable among AYAs, which is in line with observations in Belgium’s neighbouring countries [47,52]. The 5-year relative survival rate for malignant brain tumours ranges from 71% in younger AYAs to 65% in the group aged 30–39 years, largely reflecting differences in the aggressiveness of glioma subtypes by age. For example, 15% of the gliomas in AYA aged 15–29 years were glioblastomas, compared to 27% in those aged 30–39 years.

The incidence of **cervical cancer** steadily decreased from 2004 to 2020 by 1% per year. In Belgium, cervical cancer is the only cancer with a population-based screening program that includes the AYA age group, with organised screening in Flanders and opportunistic screening in Wallonia and Brussels. Since 2013, women aged 25 to 64 have been invited every three years for a cyto-histopathological screening, including human papillomavirus (HPV) triage, for equivocal results [61]. The result of this screening program is reflected in the decreasing incidence trend for cancer of genital sites, excluding ovary and testis (mostly cervix).

Where most cancer types showed decreasing/stable trends from 2004 to 2020 or in more recent years, four cancer types demonstrated a steady increase in incidence: chronic myeloid neoplasms, testis cancer, Hodgkin lymphoma, and breast cancer. These observations are aligned with those for early-onset German cancers [47] except for an increase in thyroid cancer reported there.

The adaptation to classification changes may partly explain the increased incidence trend for **chronic myeloid neoplasms** in the 2000s.

Whereas for the majority of the studied cancer types, the incidence rates increase (linearly) with age, it seems that for testis cancer and Hodgkin lymphoma, other drivers (e.g., environmental factors) influence the incidence of AYA cancer as well. Although the causal pathway of **testis cancer** is poorly understood, there is a consensus that the aetiology is multifactorial, encompassing genetic and environmental factors. Common environmental risk factors include cryptorchidism [62], family history of testicular cancer [63], infections with Epstein–Barr virus and human immunodeficiency virus [64], intrauterine exposure to high oestrogen levels [65] and smoking [66], low birth weight [67], intratubular germ cell neoplasm, and prior history of testis cancer [68]. The incidence rates of testis tumours increased significantly over the years for AYAs and older adults aged 40–49 years, which is in line with Voeltz et al. [47], who also reported an increase (Germany, early onset, 1990–2019). However, in Belgian AYAs aged 30–39 years, from 2015 onwards, no trend could be observed. These observations are aligned with the publication of Zhang et al. [46] reporting a stable trend among AYAs in the US from 2016 to 2021.

The survival rate in Belgian AYAs for **Hodgkin lymphoma (HL)** is very good for this cancer type (5-year RS is 97%); however, survivors face significant late morbidity and mortality, necessitating lifelong clinical follow-up [69,70]. The Belgian incidence rates for HL among AYAs steadily increased over the studied period, aligning with Dutch observations among AYAs (1990–2016) [52]. The Belgian incidence rate, reported by Trama et al. [4], is slightly above the European rate. Huang et al. [71] found that in the past decade, worldwide, there has been a general increase in the incidence of HL in patients younger than 40 years. The triggers for this increase remain uncertain; however, there is a hypothesis that this could be related to lifestyle, the prevalence of metabolic syndromes, and improved early detection.

**Breast cancer** incidence among Belgian female AYAs aged 15–29 years increased by 1.5% per year, whereas the trend remained stable in older AYAs (aged 30–39 years). The incidence in older adults aged 40–49 years decreased from 2004 to 2020. Van der Meer et al. [52] reported an increase in breast cancer among Dutch AYA between 1990 and 2016. The incidence of early-onset breast cancer increased in Germany as well between 1999 and 2019 [47]. AYAs are more likely than older women with breast cancer to present with unfavourable biology and advanced disease [72]. Two key reasons for age-related diagnostic delays are young women’s lack of awareness of early-onset breast cancer and the dismissal of suspicious signs as unimportant due to their age [73]. In addition, mammographic screening is not recommended for AYAs at average breast cancer risk, reflecting diagnostic delays. Hormonal and reproductive preferences risk factors for early-onset breast cancer risk include hormonal oral contraceptives [74], late parity after age 30, and a history of giving birth 5 to 10 years ago [75]. AYA breast cancer is more frequently familial, and approximately half of AYA women with breast cancer under the age of 30 have a germline predisposing mutation in *BRCA1*, *BRCA2*, or *TP53* genes [76]. A potential hypothesis is that the increased focus on more active follow-up of individuals with a familial history and/or predisposition to breast cancer may contribute to earlier diagnoses by identifying malignancies or precancerous lesions in resection specimens. Mortality rates for breast cancer in the older AYA group (aged 30–39 years) declined twice as fast as the rates for women in their forties (−3% versus −1.5% annually). Early diagnosis has likely contributed to decreased mortality from breast cancer among young women.

The **mortality** rates decreased significantly for all AYA age groups and both sexes in the studied period, 2004–2019. These findings align with observations in the Netherlands for AYAs over the period 1990–2016 [52], in Germany for early-onset cancer over 1999–2019 [47], and at the global level in AYAs over 2000–2021 [40]. These results are also in line with European trends [77]. However, rising trends in mortality for colorectal cancer among young adults were predicted for Italy, the UK, Spain, Poland, and Germany [78]. Due to low numbers, no trends for colorectal mortality could be assessed in this study. Belgium has some of the European Union’s lowest cancer mortality rates, and the five-year survival rate for the majority of cancers is higher than the European average [79]. Still, in combination with the relatively high incidence rates, the burden of cancer weighs on individuals and society [80].

The Belgian **relative survival** for AYAs is generally above the European average for multiple cancer types [9]. There is, however, room for improvement for cancers of the lung/bronchus/trachea, cancer of the gastro-intestinal tract (excl. colorectal), precursor haematopoietic neoplasms, ovarian cancer, and tumours of the central nervous system, whose 5-year RS percentages vary between 42% and 78%. The survival gap with children remains for haematological malignancies (mainly for precursor haematopoietic neoplasms), which is in line with Trama et [81], reporting that European AYA survival rates for ALL improved from 1978 to 2006 but did not reach the levels seen in children. The 5-year survival is generally better in AYAs than in older adults aged 40–49 years, except for breast cancer and sarcoma. Causes for this survival gap may be differences in disease biology, treatment, therapy-related toxicities, and psychosocial factors [82]. There is a sex difference in survival, with an overall better prognosis for female AYA. Compared to females and other age groups, the five-year RS is the lowest in male AYA aged 35–39 years. This sex difference in survival reflects the better prognosis of breast carcinomas compared to cancers diagnosed in male AYAs (such as cancers of the head and neck and cancer of the GI tract, excluding colorectal). Other variables that might lead to this survival gap between sexes are the stage of cancer at diagnosis and socioeconomic status [83]. These variables were, however, not included in the dataset.

### Strengths and Limitations

The conclusions drawn from this research stem from nearly twenty years of high-quality data, meticulously provided by registrars and physicians from Belgian oncology care programmes and laboratories for pathological anatomy and collected by skilled data managers from BCR. This study also has a number of limitations. In examining rare cancers, findings must be interpreted cautiously, as random variation due to a small number of cases can erroneously present as significant trends. In addition, low case numbers during certain sub-analyses lead to the inability to calculate average annual percentage change statistics for various cancer types. Another limitation is that it could be that the trends observed in this study may be the result of using all stages combined, whereas some trends might only become apparent with individual disease stages. Finally, due to the SEER AYA classification and the small size of some of the cancer subgroups, cancer types with heterogeneous incidence and/or survival rates had to be combined into larger categories. The international comparison was limited because of differences in exhaustivity of registration, classification used, and standardised rates.

However, the use of population-based data from BCR can be considered a major strength, as selection bias is limited due to the inclusion of the entire Belgian population. Consequently, a comprehensive depiction of trends related to incidence, survival, and mortality within the Belgian AYA population was made possible. Stratification in five-year age groups permitted a detailed assessment of age-specific trends within the AYA population, which is another major strength of this study. This facilitates the detection of substantial age-related distinctions that otherwise can be lost by grouping AYAs as a unified cohort. Moreover, the long observation period (17 years) allowed us to analyse long-term incidence and survival trends.

## 5. Conclusions

It is crucial to monitor early-onset cancer trends to determine whether the stabilising and decreasing patterns persist or if the increasing trends reverse. While it appears that some cancers are being brought under control through the reduction of known risk factors, early detection by increased awareness, and advancements in treatment, it is essential to recognize that the recent stabilisation in incidence is associated with a reduced mortality, likely due to improvements in medical care and more effective treatments. These dual trends lead to an increase in the overall cancer burden among AYAs and, subsequently, in the associated demands on healthcare resources. Given that the cancer burden is increasing in Belgium and AYAs have many years of life ahead, contributing significantly to the economy, there should be a stronger emphasis on improving prevention of AYA cancers, awareness to foster early detection, along with maintaining precise epidemiological surveillance. This group is still underrepresented in cancer research, and a clear understanding of the biological and genetic drivers of several cancers is still lacking. For addressing more complex research questions, large-scale prospective datasets with comprehensive patient, clinical, and treatment information are required. Since such data resources are limited, there must be increased efforts to initiate these collections.

Additionally, increased collaboration between paediatric and adult medical oncologists and haematologists at both national and international levels is necessary to address the distinct needs and challenges faced by this unique population. The launch of the Belgian AYA-dedicated cancer care program in November 2023 [15] made a significant step in addressing these issues.

This study is the first to show that the increased cancer burden in AYAs in Belgium is mostly due to the improved survival with a stabilised incidence in recent years and is comparable to what is observed in neighbouring countries. This study offers valuable insights to support policymakers in optimizing AYA cancer management.

## Data Availability

The cancer cohort data used and analysed during the study are available from the corresponding author on reasonable request. The pseudonymised data can be provided within the secured environment of the Belgian Cancer Registry after having been guaranteed that the applicable GDPR regulations are applied.

## Supplementary Materials

The following supporting information can be downloaded at https://www.mdpi.com/article/10.3390/cancers17091543/s1, Table S1: SEER classification, Table S2: Number of new diagnoses, Table S3: Cancer causes of death, Table S4: AAPC, Table S5: Survival, Table S6: Prevalence, Table S7: International comparison, Table S8: International comparison Classification.

## References

[1] Belgian Cancer Registry. https://kankerregister.org/default.aspx?lang=EN.

[2] (2024). StatBel. Structure of the Population Based on the National Register. https://statbel.fgov.be/.

[3] GBD 2019 Adolescent Young Adult Cancer Collaborators (2022). The Global Burden of Adolescent and Young Adult Cancer in 2019: A Systematic Analysis for the Global Burden of Disease Study 2019. Lancet Oncol..

[4] Trama A., Stark D., Bozovic-Spasojevic I., Gaspar N., Peccatori F., Toss A., Bernasconi A., Quarello P., Scheinemann K., Jezdic S. (2023). Cancer Burden in Adolescents and Young Adults in Europe. ESMO Open.

[5] Gupta S., Harper A., Ruan Y., Barr R., Frazier L., Ferlay J., Steliarova-Foucher E., Fidler-Benaoudia M. (2020). International Trends in the Incidence of Cancer Among Adolescents and Young Adults. J. Natl. Cancer Inst..

[6] International Agency for Research on Cancer (IARC), WHO Cancer Incidence in Five Continents (CI5). https://ci5.iarc.fr/ci5plus/.

[7] You L., Lv Z., Li C., Ye W., Zhou Y., Jin J., Han Q. (2021). Worldwide Cancer Statistics of Adolescents and Young Adults in 2019: A Systematic Analysis of the Global Burden of Disease Study 2019. ESMO Open.

[8] (2024). Global Burden of Disease (GBD) 2021; Institute for Health Metrics and Evaluation (IHME). http://www.healthdata.org/results/data-visualizations.

[9] Trama A., Botta L., Stiller C., Visser O., Cañete-Nieto A., Spycher B., Bielska-Lasota M., Katalinic A., Vener C., Innos K. (2024). Survival of European Adolescents and Young Adults Diagnosed with Cancer in 2010–2014. Eur. J. Cancer.

[10] Miller K.D., Fidler-Benaoudia M., Keegan T.H., Hipp H.S., Jemal A., Siegel R.L. (2020). Cancer Statistics for Adolescents and Young Adults, 2020. CA Cancer J. Clin..

[11] Wolfson J.A., Kenzik K.M., Foxworthy B., Salsman J.M., Donahue K., Nelson M., Littrell M.B., Williams G.R., Levine J.M. (2023). Understanding Causes of Inferior Outcomes in Adolescents and Young Adults with Cancer. J. Natl. Compr. Cancer Netw..

[12] Ferrari A., Stark D., Peccatori F.A., Fern L., Laurence V., Gaspar N., Bozovic-Spasojevic I., Smith O., De Munter J., Derwich K. (2021). Adolescents and Young Adults (AYA) with Cancer: A Position Paper from the AYA Working Group of the European Society for Medical Oncology (ESMO) and the European Society for Paediatric Oncology (SIOPE). ESMO Open.

[13] US Department of Health And Human Services, US National Institutes of Health, US National Cancer Institute, Adolescent and Young Adult Oncology Progress Report Group (2006). Closing the Gap: Research and Care Imperatives for Adolescents and Young Adults with Cancers. https://www.cancer.gov/types/aya/research/ayao-august-2006.pdf.

[14] Saloustros E., Stark D., Michailidou K., Mountzios G., Brugieres L., Peccatori F., Jezdic S., Essiaf S., Douillard J.-Y., Bielack S. (2017). The Care of Adolescents and Young Adults with Cancer: Results of the ESMO/SIOPE Survey. ESMO Open.

[15] Convention Entre Le Comité de l’assurance Du Service Des Soins de Santé de l’INAMI et Les Établissements Hospitaliers Pour Le Financement Des Équipes de Référence AJA Visant à Soutenir Des Soins Sur Mesure Aux AJA Atteints d’un Cancer. Note CSS 2023/321 (8 November 2023). https://www.inami.fgov.be/fr/actualites/cancer-une-prise-en-charge-sur-mesure-pour-les-adolescents-et-jeunes-adultes-grace-a-notre-financement-d-equipes-de-reference-aja.

[16] Overeenkomst Tussen Het Verzekeringscomité van de Gezondheidszorgdienst van Het RIZIV En de Ziekenhuizen Voor de Financiering van AJA-Referentieteams Met Als Doel Op Maat Gemaakte Zorg Te Ondersteunen Voor AYA’s (Adolescenten En Jongvolwassenen) Die Getroffen Zijn Door Kanker. Nota CSS 2023/321 (8 November 2023). https://www.riziv.fgov.be/nl/nieuws/zorg-op-maat-voor-adolescenten-en-jongvolwassenen-met-kanker-dankzij-onze-vergoeding-van-aya-referentieteams.

[17] Barr R., Ries L., Trama A., Gatta G., Steliarova-Foucher E., Stiller C., Bleyer A. (2020). A System for Classifying Cancers Diagnosed in Adolescents and Young Adults. Cancer.

[18] Steliarova-Foucher E., Stiller C., Lacour B., Kaatsch P. (2005). International Classification of Childhood Cancer, Third Edition. Cancer.

[19] National Cancer Institute (2020). Surveillance, Epidemiology, and End Results Program. AYA Site Recode 2020 Revision. https://seer.cancer.gov/ayarecode/aya-2020.html.

[20] Berkman A.M., Mittal N., Roth M.E. (2023). Adolescent and Young Adult Cancers: Unmet Needs and Closing the Gaps. Curr. Opin. Pediatr..

[21] McGrady M.E., Willard V.W., Williams A.M., Brinkman T.M. (2024). Psychological Outcomes in Adolescent and Young Adult Cancer Survivors. J. Clin. Oncol..

[22] (2019). Cancer in Children and Adolescents in Belgium, 2004–2016; D/2019/11.846/1; Belgian Cancer Registry. https://kankerregister.org/en/publicaties/cancer-children-and-adolescents-belgium-2004-2016.

[23] (2023). Cancer in Children and Adolescents in Belgium, 2004–2020; D/2023/11.846/1; Belgian Cancer Registry. https://kankerregister.org/en/publicaties/cancer-children-and-adolescents-belgium-2004-2020.

[24] (2025). Cancer in Adolescents and Young Adults, Belgium 2004–2022; D/2025/11.846/1; Belgian Cancer Registry: Brussels. https://kankerregister.org/en/publicaties/cancer-adolescents-and-young-adults-belgium-2004-2022.

[25] (2006). Federale Overheidsdienst Volksgezondheid, Veiligheid van de Voedselketen En Leefmilieu/Service Public Federal Sante Publique, Securite de La Chaine Alimentaire et Environnement. Wet Houdende Diverse Bepalingen Betreffende Gezondheid/Loi Portant Dispositions Diverses En Matière de Santé. https://etaamb.openjustice.be/nl/wet-van-13-december-2006_n2006023386.html.

[26] Service Public Federal Sante Publique, Securite de La Chaine Alimentaire et Environnement. Loi Portant Dispositions Diverses En Matière deSanté. https://etaamb.openjustice.be/fr/loi-du-13-decembre-2006_n2006023386.

[27] Statbel Mortality, Life Expectancy and Causes of Death.; 2023. https://statbel.fgov.be/en/themes/population/mortality-life-expectancy-and-causes-death.

[28] ESMO/SIOPE Adolescents and Young Adults Working Group. https://www.esmo.org/about-esmo/organisational-structure/educational-committee/adolescents-and-young-adults-working-group.

[29] Centraal Bureau voor de Statistiek (CBS). https://www.cbs.nl/nl-nl.

[30] Integraal Kankercentrum Nederlands (IKNL). https://nkr-cijfers.iknl.nl/.

[31] Institut National de la Statistique et des Etudes Economiques (Insee). https://www.insee.fr/.

[32] Institut National du Cancer. https://www.e-cancer.fr/.

[33] DeStatis Statistisches Bundesamt. https://www.destatis.de/EN/Themes/Society-Environment/Population/.

[34] Zentrum Für Krebsregisterdaten, Robert Koch Institut. https://www.krebsdaten.de/Krebs/.

[35] (2013). Eurostat’s Task Force, M. and W. Papers. Revision of the European Standard Population. https://ec.europa.eu/eurostat/documents/3859598/5926869/KS-RA-13-028-EN.PDF.pdf/e713fa79-1add-44e8-b23d-5e8fa09b3f8f?t=1414782757000.

[36] Ahmad O.B., Boschi Pinto C., Lopez A.D., Murray C.J., Lozano R., Inoue M. (2001). Age Standardization of Rates: A New WHO Standard.

[37] Ederer F., Axtell L., Cutler S. (1961). The Relative Survival Rate: A Statistical Methodology. Natl. Cancer Inst. Monogr..

[38] (2023). Joinpoint Regression Program, Version 5.0.2. https://surveillance.cancer.gov/joinpoint/.

[39] National Cancer Institute—Surveillance Research Program. Number of Joinpoints—Joinpoint Help System. https://surveillance.cancer.gov/help/joinpoint/setting-parameters/advanced-tab/number-of-joinpoints.

[40] Li W., Liang H., Wang W., Liu J., Liu X., Lao S., Liang W., He J. (2024). Global Cancer Statistics for Adolescents and Young Adults: Population Based Study. J. Hematol. Oncol..

[41] Zhao J., Xu L., Sun J., Song M., Wang L., Yuan S., Zhu Y., Wan Z., Larsson S., Tsilidis K. (2023). Global Trends in Incidence, Death, Burden and Risk Factors of Early-Onset Cancer from 1990 to 2019. BMJ Oncol..

[42] Ugai T., Sasamoto N., Lee H.-Y., Ando M., Song M., Tamimi R.M., Kawachi I., Campbell P.T., Giovannucci E.L., Weiderpass E. (2022). Is Early-Onset Cancer an Emerging Global Epidemic? Current Evidence and Future Implications. Nat. Rev. Clin. Oncol..

[43] Toss A., Quarello P., Mascarin M., Banna G.L., Zecca M., Cinieri S., Peccatori F.A., Ferrari A. (2022). Cancer Predisposition Genes in Adolescents and Young Adults (AYAs): A Review Paper from the Italian AYA Working Group. Curr. Oncol. Rep..

[44] Sung H., Siegel R.L., Hyun N., Miller K.D., Yabroff K.R., Jemal A. (2022). Subsequent Primary Cancer Risk Among 5-Year Survivors of Adolescent and Young Adult Cancers. JNCI J. Natl. Cancer Inst..

[45] Macq G., Silversmit G., Verdoodt F., Van Eycken E. (2023). The Epidemiology of Multiple Primary Cancers in Belgium (2004–2017): Incidence, Proportion, Risk, Stage and Impact on Relative Survival Estimates. BMC Cancer.

[46] Zhang L., Muscat J.E., Chinchilli V.M., Behura C.G. (2024). Trends in Cancer Incidence and Mortality in US Adolescents and Young Adults, 2016–2021. Cancers.

[47] Voeltz D., Baginski K., Hornberg C., Hoyer A. (2024). Trends in Incidence and Mortality of Early-Onset Cancer in Germany between 1999 and 2019. Eur. J. Epidemiol..

[48] An S., Kim K., Moon S., Ko K.-P., Kim I., Lee J., Park S. (2021). Indoor Tanning and the Risk of Overall and Early-Onset Melanoma and Non-Melanoma Skin Cancer: Systematic Review and Meta-Analysis. Cancers.

[49] Del Marmol V. (2022). Prevention and Screening of Melanoma in Europe: 20 Years of the Euromelanoma Campaign. J. Eur. Acad. Dermatol. Venereol..

[50] Stichting Tegen Kanker—Fondation Contre le Cancer Ondanks preventie neemt huidkanker toe—Malgré la prévention, les cancers de la peau sont en hausse. https://kanker.be/resource/ondanks-preventie-neemt-huidkanker-toe/-https://cancer.be/ressource/malgre-la-prevention-les-cancers-de-la-peau-sont-en-hausse/.

[51] LeClair K., Bell K., Furuya-Kanamori L., Doi S., Francis D. (2021). Evaluation of Gender Inequity in Thyroid Cancer Diagnosis: Differences by Sex in US Thyroid Cancer Incidence Compared with a Meta-Analysis of Subclinical Thyroid Cancer Rates at Autopsy. JAMA Internal Medicine.

[52] van der Meer D.J., Karim-Kos H.E., van der Mark M., Aben K.K., Bijlsma R.M., Rijneveld A.W., van der Graaf W.T., Husson O. (2020). Incidence, Survival, and Mortality Trends of Cancers Diagnosed in Adolescents and Young Adults (15–39 Years): A Population-Based Study in The Netherlands 1990–2016. Cancers.

[53] Kitahara C.M., Schneider A.B. (2022). Epidemiology of Thyroid Cancer. Cancer Epidemiol. Biomark. Prev..

[54] Zaridze D., Maximovitch D., Smans M., Stilidi I. (2021). Thyroid Cancer Overdiagnosis Revisited. Cancer Epidemiol..

[55] Van den Bruel A., Francart J., Dubois C., Adam M., Vlayen J., De Schutter H., Stordeur S., Decallonne B. (2013). Regional Variation in Thyroid Cancer Incidence in Belgium Is Associated with Variation in Thyroid Imaging and Thyroid Disease Management. J. Clin. Endocrinol. Metab..

[56] Frostberg E., Rahr H.B. (2020). Clinical Characteristics and a Rising Incidence of Early-Onset Colorectal Cancer in a Nationwide Cohort of 521 Patients Aged 18-40 Years. Cancer Epidemiol..

[57] Vuik F.E., Nieuwenburg S.A., Bardou M., Lansdorp-Vogelaar I., Dinis-Ribeiro M., Bento M.J., Zadnik V., Pellisé M., Esteban L., Kaminski M.F. (2019). Increasing Incidence of Colorectal Cancer in Young Adults in Europe over the Last 25 Years. Gut.

[58] Danial D., Youssef E.D., Maryam B.M., Mohammad A., Moein B.M., Liliane D. (2022). Risk Factors of Young-Onset Colorectal Cancer: Analysis of a Large Population-Based Registry. Can. J. Gastroenterol. Hepatol..

[59] Hua H., Jiang Q., Sun P., Xu X. (2023). Risk Factors for Early-Onset Colorectal Cancer: Systematic Review and Meta-Analysis. Front. Oncol..

[60] Lawler T., Parlato L., Warren Andersen S. (2024). The Histological and Molecular Characteristics of Early-Onset Colorectal Cancer: A Systematic Review and Meta-Analysis. Front. Oncol..

[61] Cervical Cancer Screening Program. https://baarmoederhalskanker.bevolkingsonderzoek.be/en.

[62] Landero-Huerta D.A., Vigueras-Villaseñor R.M., Yokoyama-Rebollar E., García-Andrade F., Rojas-Castañeda J.C., Herrera-Montalvo L.A., Díaz-Chávez J., Pérez-Añorve I.X., Aréchaga-Ocampo E., Chávez-Saldaña M.D. (2020). Cryptorchidism and Testicular Tumor: Comprehensive Analysis of Common Clinical Features and Search of SNVs in the KIT and AR Genes. Front. Cell Dev. Biol..

[63] Del Risco Kollerud R., Ruud E., Haugnes H.S., Cannon-Albright L.A., Thoresen M., Nafstad P., Vlatkovic L., Blaasaas K.G., Næss Ø., Claussen B. (2019). Family History of Cancer and Risk of Paediatric and Young Adult’s Testicular Cancer: A Norwegian Cohort Study. Br. J. Cancer.

[64] Garolla A., Vitagliano A., Muscianisi F., Valente U., Ghezzi M., Andrisani A., Ambrosini G., Foresta C. (2019). Role of Viral Infections in Testicular Cancer Etiology: Evidence From a Systematic Review and Meta-Analysis. Front. Endocrinol..

[65] Fénichel P., Chevalier N. (2019). Is Testicular Germ Cell Cancer Estrogen Dependent? The Role of Endocrine Disrupting Chemicals. Endocrinology.

[66] Hermansen M., Hjelmborg J., Thinggaard M., Znaor A., Skakkebæk N.E., Lindahl-Jacobsen R. (2025). Smoking and Testicular Cancer: A Danish Nationwide Cohort Study. Cancer Epidemiol..

[67] Chen C., Chen X., Wu D., Wang H., Wang C., Shen J., An Y., Zhong R., Li C., Liang W. (2023). Association of Birth Weight with Cancer Risk: A Dose–Response Meta-Analysis and Mendelian Randomization Study. J. Cancer Res. Clin. Oncol..

[68] Gaddam S.J., Chestnut G.T. (2023). Testicle Cancer.

[69] Lo A.C., Chen B., Samuel V., Savage K.J., Freeman C., Goddard K. (2021). Late Effects in Survivors Treated for Lymphoma as Adolescents and Young Adults: A Population-Based Analysis. J. Cancer Surviv..

[70] Xavier A.C., Epperla N., Taub J.W., Costa L.J. (2018). Excess Mortality among 10-Year Survivors of Classical Hodgkin Lymphoma in Adolescents and Young Adults. Am. J. Hematol..

[71] Huang J., Pang W.S., Lok V., Zhang L., Lucero-Prisno D.E., Xu W., Zheng Z.-J., Elcarte E., Withers M., Wong M.C.S. (2022). Incidence, Mortality, Risk Factors, and Trends for Hodgkin Lymphoma: A Global Data Analysis. J. Hematol. Oncol..

[72] Murphy B.L., Day C.N., Hoskin T.L., Habermann E.B., Boughey J.C. (2019). Adolescents and Young Adults with Breast Cancer Have More Aggressive Disease and Treatment than Patients in Their Forties. Ann. Surg. Oncol..

[73] Kumar R., Saini S., Ganguly N.K. (2023). Year-Round Breast Cancer Awareness: Empowering Young Women in the Fight against Breast Cancer. Indian J. Med. Res..

[74] Hultstrand J.N., Gemzell-Danielsson K., Kallner H.K., Lindman H., Wikman P., Sundström-Poromaa I. (2022). Hormonal Contraception and Risk of Breast Cancer and Breast Cancer in Situ among Swedish Women 15–34 Years of Age: A Nationwide Register-Based Study. Lancet Reg. Health—Eur..

[75] Nichols H.B., Schoemaker M.J., Cai J., Xu J., Wright L.B., Brook M.N., Jones M.E., Adami H.O., Baglietto L., Bertrand K.A. (2019). Breast Cancer Risk After Recent Childbirth: A Pooled Analysis of 15 Prospective Studies. Ann. Intern. Med..

[76] Lalloo F., Varley J., Moran A., Ellis D., O’dair L., Pharoah P., Antoniou A., Hartley R., Shenton A., Seal S. (2006). BRCA1, BRCA2 and TP53 Mutations in Very Early-Onset Breast Cancer with Associated Risks to Relatives. Eur. J. Cancer.

[77] Santucci C., Mignozzi S., Alicandro G., Pizzato M., Malvezzi M., Negri E., Jha P., La Vecchia C. (2025). Trends in Cancer Mortality under Age 50 in 15 Upper-Middle and High-Income Countries. JNCI J. Natl. Cancer Inst..

[78] Malvezzi M., Carioli G., Bertuccio P., Boffetta P., Levi F., La Vecchia C., Negri E. (2024). European Cancer Mortality Predictions for the Year 2024 with Focus on Colorectal Cancer. Ann. Oncol..

[79] OECD Publishing (2023). EU Country Cancer Profile: Belgium 2023, EU Country Cancer Profiles.

[80] BeBOD—Belgian National Burden of Disease Study; Sciensano. https://www.sciensano.be/en/projects/belgian-national-burden-disease-study.

[81] Trama A., Bernasconi A., McCabe M.G., Guevara M., Gatta G., Botta L., Ries L., Bleyer A., the RARECAREnet Working Group (2019). Is the Cancer Survival Improvement in European and American Adolescent and Young Adults Still Lagging behind That in Children?. Pediatr. Blood Cancer.

[82] Keegan T.H.M., Ries L.A.G., Barr R.D., Geiger A.M., Dahlke D.V., Pollock B.H., Bleyer W.A., National Cancer Institute Next Steps for Adolescent and Young Adult Oncology Epidemiology Working Group (2016). Comparison of Cancer Survival Trends in the United States of Adolescents and Young Adults with Those in Children and Older Adults. Cancer.

[83] Moke D., Tsai K.-Y., Hamilton A., Hwang A., Liu L., Freyer D., Deapen D. (2019). Emerging Cancer Survival Trends, Disparities, and Priorities in Adolescents and Young Adults: A California Cancer Registry-Based Study. JNCI Cancer Spectr..

