# Supplementary material for: Cancer Burden in Adolescents and Young Adults in Belgium: Trends to Incidence Stabilisation in Recent Years with Improved Survival
Source: Cancers (Basel). 2025 May 1;17(9):1543. doi: 10.3390/cancers17091543 (PMC12071148; doi:10.3390/cancers17091543)
Supplement: Supplementary file 1 [file cancers-17-01543-s001.zip › Table S2 Number of new diagnoses.pdf]

Table S2. Number of new diagnoses, crude rate, and age-adjusted incidence rates of patients, aged 15-39 years at the time of diagnosis in Belgium, 2004-2020.

Age group	N	CR	ESR 1976	ESR 1976 [95% CI]	ESR 2013	ESR 2013 [95% CI]	WSR	WSR [95% CI]
**Males**								
5-14 years	1,487	13.7	13.7	[13,0;14,4]	13.7	[13,0;14,4]	13.7	[13,0;14,4]
15-19 years	1,385	25.2	25.2	[23,8;26,5]	25.2	[23,8;26,5]	25.2	[23,8;26,5]
20-24 years	2,146	37.6	37.6	[36,0;39,2]	37.6	[36,0;39,2]	37.6	[36,0;39,2]
25-29 years	3,214	54.0	54.0	[52,1;55,8]	54.0	[52,1;55,8]	54.0	[52,1;55,8]
30-34 years	4,206	68.6	68.6	[66,6;70,7]	68.6	[66,6;70,7]	68.6	[66,6;70,7]
35-39 years	5,709	89.0	89.0	[86,7;91,3]	89.0	[86,7;91,3]	89.0	[86,7;91,3]
40-49 years	26,288	194.4	193.8	[191,5;196,2]	193.8	[191,5;196,2]	193.8	[191,5;196,2]
15-39 years	16,660	56.1	54.9	[54,0;55,7]	56.7	[55,8;57,5]	51.5	[50,7;52,3]
**Females**								
5-14 years	1,187	11.5	11.5	[10,8;12,1]	11.5	[10,8;12,1]	11.4	[10,7;12,0]
15-19 years	1,165	22.1	22.1	[20,8;23,4]	22.1	[20,8;23,4]	22.1	[20,8;23,4]
20-24 years	2,174	38.7	38.7	[37,0;40,3]	38.7	[37,0;40,3]	38.7	[37,0;40,3]
25-29 years	4,084	68.6	68.6	[66,5;70,7]	68.6	[66,5;70,7]	68.6	[66,5;70,7]
30-34 years	7,095	116.7	116.7	[114,0;119,4]	116.7	[114,0;119,4]	116.7	[114,0;119,4]
35-39 years	12,357	196.2	196.2	[192,7;199,6]	196.2	[192,7;199,6]	196.2	[192,7;199,6]
40-49 years	53,151	401.9	400.9	[397,5;404,3]	400.9	[397,5;404,3]	400.9	[397,5;404,3]
15-39 years	26,875	92.0	88.5	[87,4;89,5]	93.5	[92,3;94,6]	79.3	[78,3;80,3]
**Both sexes**								
5-14 years	2,674	12.6	12.6	[12,1;13,1]	12.6	[12,1;13,1]	12.5	[12,1;13,0]
15-19 years	2,550	23.7	23.7	[22,7;24,6]	23.7	[22,7;24,6]	23.7	[22,7;24,6]
20-24 years	4,320	38.1	38.1	[37,0;39,2]	38.1	[37,0;39,2]	38.1	[37,0;39,2]
25-29 years	7,298	61.3	61.3	[59,9;62,7]	61.3	[59,9;62,7]	61.3	[59,9;62,7]
30-34 years	11,301	92.6	92.6	[90,9;94,3]	92.6	[90,9;94,3]	92.6	[90,9;94,3]
35-39 years	18,066	142.1	142.1	[140,1;144,2]	142.1	[140,1;144,2]	142.1	[140,1;144,2]
40-49 years	79,439	297.0	296.2	[294,2;298,3]	296.2	[294,2;298,3]	296.2	[294,2;298,3]
15-39 years	43,535	73.9	71.6	[70,9;72,2]	74.9	[74,2;75,6]	65.3	[64,7;65,9]
**Hodgkin Lymphoma**								
5-14 years	235	1.11	1.11	[1,0;1,3]	1.11	[1,0;1,3]	1.08	[0,9;1,2]
15-19 years	502	4.66	4.66	[4,2;5,1]	4.66	[4,2;5,1]	4.66	[4,2;5,1]
20-24 years	629	5.55	5.55	[5,1;6,0]	5.55	[5,1;6,0]	5.55	[5,1;6,0]
25-29 years	576	4.84	4.84	[4,4;5,2]	4.84	[4,4;5,2]	4.84	[4,4;5,2]
30-34 years	473	3.87	3.87	[3,5;4,2]	3.87	[3,5;4,2]	3.87	[3,5;4,2]
35-39 years	430	3.38	3.38	[3,1;3,7]	3.38	[3,1;3,7]	3.38	[3,1;3,7]
40-49 years	678	2.53	2.54	[2,3;2,7]	2.54	[2,3;2,7]	2.54	[2,3;2,7]
15-39 years	2,610	4.43	4.46	[4,3;4,6]	4.41	[4,2;4,6]	4.56	[4,4;4,7]
**Mature B-cell neoplasms**								
5-14 years	184	0.9	0.9	[0,7;1,0]	0.9	[0,7;1,0]	0.9	[0,7;1,0]
15-19 years	152	1.4	1.4	[1,2;1,6]	1.4	[1,2;1,6]	1.4	[1,2;1,6]
20-24 years	206	1.8	1.8	[1,6;2,1]	1.8	[1,6;2,1]	1.8	[1,6;2,1]
25-29 years	288	2.4	2.4	[2,1;2,7]	2.4	[2,1;2,7]	2.4	[2,1;2,7]
30-34 years	460	3.8	3.8	[3,4;4,1]	3.8	[3,4;4,1]	3.8	[3,4;4,1]
35-39 years	739	5.8	5.8	[5,4;6,2]	5.8	[5,4;6,2]	5.8	[5,4;6,2]
40-49 years	3,575	13.4	13.3	[12,9;13,8]	13.3	[12,9;13,8]	13.3	[12,9;13,8]
15-39 years	1,845	3.1	3.0	[2,9;3,2]	3.2	[3,0;3,3]	2.8	[2,7;2,9]
**Mature T-cell and NK-cell neoplasms**								
5-14 years	48	0.2	0.2	[0,2;0,3]	0.2	[0,2;0,3]	0.2	[0,2;0,3]
15-19 years	43	0.4	0.4	[0,3;0,5]	0.4	[0,3;0,5]	0.4	[0,3;0,5]
20-24 years	58	0.5	0.5	[0,4;0,6]	0.5	[0,4;0,6]	0.5	[0,4;0,6]
25-29 years	85	0.7	0.7	[0,6;0,9]	0.7	[0,6;0,9]	0.7	[0,6;0,9]
30-34 years	128	1.0	1.0	[0,9;1,2]	1.0	[0,9;1,2]	1.0	[0,9;1,2]
35-39 years	136	1.1	1.1	[0,9;1,2]	1.1	[0,9;1,2]	1.1	[0,9;1,2]
40-49 years	411	1.5	1.5	[1,4;1,7]	1.5	[1,4;1,7]	1.5	[1,4;1,7]
15-39 years	450	0.8	0.7	[0,7;0,8]	0.8	[0,7;0,8]	0.7	[0,6;0,8]
**Other lymphoid neoplasms**								
5-14 years	8	0.0	0.0	[0,0;0,1]	0.0	[0,0;0,1]	0.0	[0,0;0,1]
15-19 years	16	0.1	0.1	[0,1;0,2]	0.1	[0,1;0,2]	0.1	[0,1;0,2]

20-24 years	22	0.2	0.2	[0,1;0,3]	0.2	[0,1;0,3]	0.2	[0,1;0,3]
25-29 years	24	0.2	0.2	[0,1;0,3]	0.2	[0,1;0,3]	0.2	[0,1;0,3]
30-34 years	21	0.2	0.2	[0,1;0,2]	0.2	[0,1;0,2]	0.2	[0,1;0,2]
35-39 years	28	0.2	0.2	[0,1;0,3]	0.2	[0,1;0,3]	0.2	[0,1;0,3]
40-49 years	112	0.4	0.4	[0,3;0,5]	0.4	[0,3;0,5]	0.4	[0,3;0,5]
15-39 years	111	0.2	0.2	[0,2;0,2]	0.2	[0,2;0,2]	0.2	[0,2;0,2]
**Precursor hematopoietic neoplasms**								
5-14 years	669	3.2	3.2	[2,9;3,4]	3.2	[2,9;3,4]	3.2	[2,9;3,4]
15-19 years	286	2.7	2.7	[2,3;3,0]	2.7	[2,3;3,0]	2.7	[2,3;3,0]
20-24 years	225	2.0	2.0	[1,7;2,2]	2.0	[1,7;2,2]	2.0	[1,7;2,2]
25-29 years	214	1.8	1.8	[1,6;2,0]	1.8	[1,6;2,0]	1.8	[1,6;2,0]
30-34 years	270	2.2	2.2	[1,9;2,5]	2.2	[1,9;2,5]	2.2	[1,9;2,5]
35-39 years	298	2.3	2.3	[2,1;2,6]	2.3	[2,1;2,6]	2.3	[2,1;2,6]
40-49 years	851	3.2	3.2	[3,0;3,4]	3.2	[3,0;3,4]	3.2	[3,0;3,4]
15-39 years	1,293	2.2	2.2	[2,1;2,3]	2.2	[2,1;2,3]	2.2	[2,1;2,3]
**Chronic myeloid neoplasms**								
5-14 years	103	0.5	0.5	[0,4;0,6]	0.5	[0,4;0,6]	0.5	[0,4;0,6]
15-19 years	81	0.8	0.8	[0,6;0,9]	0.8	[0,6;0,9]	0.8	[0,6;0,9]
20-24 years	132	1.2	1.2	[1,0;1,4]	1.2	[1,0;1,4]	1.2	[1,0;1,4]
25-29 years	212	1.8	1.8	[1,5;2,0]	1.8	[1,5;2,0]	1.8	[1,5;2,0]
30-34 years	297	2.4	2.4	[2,2;2,7]	2.4	[2,2;2,7]	2.4	[2,2;2,7]
35-39 years	434	3.4	3.4	[3,1;3,7]	3.4	[3,1;3,7]	3.4	[3,1;3,7]
40-49 years	1,555	5.8	5.8	[5,5;6,1]	5.8	[5,5;6,1]	5.8	[5,5;6,1]
15-39 years	1,156	2.0	1.9	[1,8;2,0]	2.0	[1,9;2,1]	1.8	[1,7;1,9]
**Histiocytic and dendritic cell neoplasms (HDCN)**								
5-14 years	96	0.5	0.5	[0,4;0,5]	0.5	[0,4;0,5]	0.5	[0,4;0,5]
15-19 years	27	0.3	0.3	[0,2;0,3]	0.3	[0,2;0,3]	0.3	[0,2;0,3]
20-24 years	21	0.2	0.2	[0,1;0,3]	0.2	[0,1;0,3]	0.2	[0,1;0,3]
25-29 years	23	0.2	0.2	[0,1;0,3]	0.2	[0,1;0,3]	0.2	[0,1;0,3]
30-34 years	23	0.2	0.2	[0,1;0,3]	0.2	[0,1;0,3]	0.2	[0,1;0,3]
35-39 years	42	0.3	0.3	[0,2;0,4]	0.3	[0,2;0,4]	0.3	[0,2;0,4]
40-49 years	68	0.3	0.3	[0,2;0,3]	0.3	[0,2;0,3]	0.3	[0,2;0,3]
15-39 years	136	0.2	0.2	[0,2;0,3]	0.2	[0,2;0,3]	0.2	[0,2;0,3]
**CNS**								
5-14 years	484	2.3	2.3	[2,1;2,5]	2.3	[2,1;2,5]	2.3	[2,1;2,5]
15-19 years	199	1.8	1.8	[1,6;2,1]	1.8	[1,6;2,1]	1.8	[1,6;2,1]
20-24 years	274	2.4	2.4	[2,1;2,7]	2.4	[2,1;2,7]	2.4	[2,1;2,7]
25-29 years	411	3.5	3.5	[3,1;3,8]	3.5	[3,1;3,8]	3.5	[3,1;3,8]
30-34 years	484	4.0	4.0	[3,6;4,3]	4.0	[3,6;4,3]	4.0	[3,6;4,3]
35-39 years	563	4.4	4.4	[4,1;4,8]	4.4	[4,1;4,8]	4.4	[4,1;4,8]
40-49 years	1,761	6.6	6.6	[6,3;6,9]	6.6	[6,3;6,9]	6.6	[6,3;6,9]
15-39 years	1,931	3.3	3.2	[3,1;3,4]	3.3	[3,1;3,4]	3.1	[2,9;3,2]
**Sarcoma**								
5-14 years	417	2.0	2.0	[1,8;2,2]	2.0	[1,8;2,2]	1.9	[1,7;2,1]
15-19 years	378	3.5	3.5	[3,2;3,9]	3.5	[3,2;3,9]	3.5	[3,2;3,9]
20-24 years	319	2.8	2.8	[2,5;3,1]	2.8	[2,5;3,1]	2.8	[2,5;3,1]
25-29 years	402	3.4	3.4	[3,0;3,7]	3.4	[3,0;3,7]	3.4	[3,0;3,7]
30-34 years	511	4.2	4.2	[3,8;4,5]	4.2	[3,8;4,5]	4.2	[3,8;4,5]
35-39 years	679	5.3	5.3	[4,9;5,7]	5.3	[4,9;5,7]	5.3	[4,9;5,7]
40-49 years	2,124	7.9	7.9	[7,6;8,3]	7.9	[7,6;8,3]	7.9	[7,6;8,3]
15-39 years	2,289	3.9	3.8	[3,7;4,0]	3.9	[3,7;4,1]	3.7	[3,6;3,9]
**Skin melanoma**								
5-14 years	46	0.2	0.2	[0,2;0,3]	0.2	[0,2;0,3]	0.2	[0,1;0,3]
15-19 years	165	1.5	1.5	[1,3;1,8]	1.5	[1,3;1,8]	1.5	[1,3;1,8]
20-24 years	627	5.5	5.5	[5,1;6,0]	5.5	[5,1;6,0]	5.5	[5,1;6,0]
25-29 years	1,198	10.1	10.1	[9,5;10,6]	10.1	[9,5;10,6]	10.1	[9,5;10,6]
30-34 years	1,727	14.1	14.1	[13,5;14,8]	14.1	[13,5;14,8]	14.1	[13,5;14,8]
35-39 years	2,470	19.4	19.4	[18,7;20,2]	19.4	[18,7;20,2]	19.4	[18,7;20,2]
40-49 years	7,225	27.0	27.0	[26,4;27,6]	27.0	[26,4;27,6]	27.0	[26,4;27,6]
15-39 years	6,187	10.5	10.1	[9,9;10,4]	10.6	[10,4;10,9]	9.2	[9,0;9,4]
**All Gonadal and related tumors**								

5-14 years	97	0.5	0.5	[0,4;0,5]	0.5	[0,4;0,5]	0.4	[0,4;0,5]
15-19 years	283	2.6	2.6	[2,3;2,9]	2.6	[2,3;2,9]	2.6	[2,3;2,9]
20-24 years	757	6.7	6.7	[6,2;7,2]	6.7	[6,2;7,2]	6.7	[6,2;7,2]
25-29 years	1,277	10.7	10.7	[10,1;11,3]	10.7	[10,1;11,3]	10.7	[10,1;11,3]
30-34 years	1,373	11.2	11.2	[10,7;11,8]	11.2	[10,7;11,8]	11.2	[10,7;11,8]
35-39 years	1,247	9.8	9.8	[9,3;10,4]	9.8	[9,3;10,4]	9.8	[9,3;10,4]
40-49 years	2,298	8.6	8.6	[8,2;8,9]	8.6	[8,2;8,9]	8.6	[8,2;8,9]
15-39 years	4,937	8.4	8.2	[8,0;8,4]	8.4	[8,2;8,6]	7.8	[7,6;8,0]
**Testis**								
5-14 years	4	0.0	0.0	[0,0;0,0]	0.0	[0,0;0,0]	0.0	[0,0;0,0]
15-19 years	195	1.8	1.8	[1,6;2,1]	1.8	[1,6;2,1]	1.8	[1,6;2,1]
20-24 years	636	5.6	5.6	[5,2;6,0]	5.6	[5,2;6,0]	5.6	[5,2;6,0]
25-29 years	1,087	9.1	9.1	[8,6;9,7]	9.1	[8,6;9,7]	9.1	[8,6;9,7]
30-34 years	1,188	9.7	9.7	[9,2;10,3]	9.7	[9,2;10,3]	9.7	[9,2;10,3]
35-39 years	955	7.5	7.5	[7,0;8,0]	7.5	[7,0;8,0]	7.5	[7,0;8,0]
40-49 years	1,118	4.2	4.2	[3,9;4,4]	4.2	[3,9;4,4]	4.2	[3,9;4,4]
15-39 years	4,061	6.9	6.8	[6,6;7,0]	6.9	[6,7;7,1]	6.4	[6,2;6,6]
**Ovary**								
5-14 years	45	0.2	0.2	[0,2;0,3]	0.2	[0,2;0,3]	0.2	[0,1;0,3]
15-19 years	51	0.5	0.5	[0,3;0,6]	0.5	[0,3;0,6]	0.5	[0,3;0,6]
20-24 years	73	0.6	0.6	[0,5;0,8]	0.6	[0,5;0,8]	0.6	[0,5;0,8]
25-29 years	124	1.0	1.0	[0,9;1,2]	1.0	[0,9;1,2]	1.0	[0,9;1,2]
30-34 years	127	1.0	1.0	[0,9;1,2]	1.0	[0,9;1,2]	1.0	[0,9;1,2]
35-39 years	242	1.9	1.9	[1,7;2,1]	1.9	[1,7;2,1]	1.9	[1,7;2,1]
40-49 years	1,116	4.2	4.2	[3,9;4,4]	4.2	[3,9;4,4]	4.2	[3,9;4,4]
15-39 years	617	1.0	1.0	[0,9;1,1]	1.1	[1,0;1,1]	1.0	[0,9;1,0]
**Germ cell and trophoblastic – CNS**								
5-14 years	44	0.2	0.2	[0,1;0,3]	0.2	[0,1;0,3]	0.2	[0,1;0,3]
15-19 years	25	0.2	0.2	[0,1;0,3]	0.2	[0,1;0,3]	0.2	[0,1;0,3]
20-24 years	24	0.2	0.2	[0,1;0,3]	0.2	[0,1;0,3]	0.2	[0,1;0,3]
25-29 years	17	0.1	0.1	[0,1;0,2]	0.1	[0,1;0,2]	0.1	[0,1;0,2]
30-34 years	9	0.1	0.1	[0,0;0,1]	0.1	[0,0;0,1]	0.1	[0,0;0,1]
35-39 years	5	0.0	0.0	[0,0;0,1]	0.0	[0,0;0,1]	0.0	[0,0;0,1]
40-49 years	13	0.0	0.0	[0,0;0,1]	0.0	[0,0;0,1]	0.0	[0,0;0,1]
15-39 years	80	0.1	0.1	[0,1;0,2]	0.1	[0,1;0,2]	0.2	[0,1;0,2]
**Germ cell and trophoblastic excluding CNS, ovary, testis**								
5-14 years	4	0.0	0.0	[0,0;0,0]	0.0	[0,0;0,0]	0.0	[0,0;0,0]
15-19 years	11	0.1	0.1	[0,0;0,2]	0.1	[0,0;0,2]	0.1	[0,0;0,2]
20-24 years	24	0.2	0.2	[0,1;0,3]	0.2	[0,1;0,3]	0.2	[0,1;0,3]
25-29 years	49	0.4	0.4	[0,3;0,5]	0.4	[0,3;0,5]	0.4	[0,3;0,5]
30-34 years	49	0.4	0.4	[0,3;0,5]	0.4	[0,3;0,5]	0.4	[0,3;0,5]
35-39 years	44	0.3	0.3	[0,2;0,4]	0.3	[0,2;0,4]	0.3	[0,2;0,4]
40-49 years	46	0.2	0.2	[0,1;0,2]	0.2	[0,1;0,2]	0.2	[0,1;0,2]
15-39 years	177	0.3	0.3	[0,3;0,3]	0.3	[0,3;0,3]	0.3	[0,2;0,3]
**Thyroid carcinoma**								
5-14 years	63	0.3	0.3	[0,2;0,4]	0.3	[0,2;0,4]	0.3	[0,2;0,4]
15-19 years	149	1.4	1.4	[1,2;1,6]	1.4	[1,2;1,6]	1.4	[1,2;1,6]
20-24 years	393	3.5	3.5	[3,1;3,8]	3.5	[3,1;3,8]	3.5	[3,1;3,8]
25-29 years	668	5.6	5.6	[5,2;6,0]	5.6	[5,2;6,0]	5.6	[5,2;6,0]
30-34 years	931	7.6	7.6	[7,1;8,1]	7.6	[7,1;8,1]	7.6	[7,1;8,1]
35-39 years	1,286	10.1	10.1	[9,6;10,7]	10.1	[9,6;10,7]	10.1	[9,6;10,7]
40-49 years	3,154	11.8	11.8	[11,4;12,2]	11.8	[11,4;12,2]	11.8	[11,4;12,2]
15-39 years	3,427	5.8	5.6	[5,5;5,8]	5.9	[5,7;6,1]	5.2	[5,0;5,4]
**Other carcinoma of head and neck**								
5-14 years	32	0.2	0.2	[0,1;0,2]	0.2	[0,1;0,2]	0.1	[0,1;0,2]
15-19 years	38	0.4	0.4	[0,2;0,5]	0.4	[0,2;0,5]	0.4	[0,2;0,5]
20-24 years	84	0.7	0.7	[0,6;0,9]	0.7	[0,6;0,9]	0.7	[0,6;0,9]
25-29 years	128	1.1	1.1	[0,9;1,3]	1.1	[0,9;1,3]	1.1	[0,9;1,3]
30-34 years	223	1.8	1.8	[1,6;2,1]	1.8	[1,6;2,1]	1.8	[1,6;2,1]
35-39 years	371	2.9	2.9	[2,6;3,2]	2.9	[2,6;3,2]	2.9	[2,6;3,2]
40-49 years	3,535	13.2	13.2	[12,7;13,6]	13.2	[12,7;13,6]	13.2	[12,7;13,6]

15-39 years	844	1.4	1.4	[1,3;1,5]	1.5	[1,4;1,6]	1.2	[1,2;1,3]
**Carcinoma of gastrointestinal tract**								
5-14 years	153	0.7	0.7	[0,6;0,8]	0.7	[0,6;0,8]	0.7	[0,6;0,8]
15-19 years	221	2.1	2.1	[1,8;2,3]	2.1	[1,8;2,3]	2.1	[1,8;2,3]
20-24 years	358	3.2	3.2	[2,8;3,5]	3.2	[2,8;3,5]	3.2	[2,8;3,5]
25-29 years	534	4.5	4.5	[4,1;4,9]	4.5	[4,1;4,9]	4.5	[4,1;4,9]
30-34 years	945	7.7	7.7	[7,2;8,2]	7.7	[7,2;8,2]	7.7	[7,2;8,2]
35-39 years	1,694	13.3	13.3	[12,7;14,0]	13.3	[12,7;14,0]	13.3	[12,7;14,0]
40-49 years	9,740	36.4	36.3	[35,6;37,0]	36.3	[35,6;37,0]	36.3	[35,6;37,0]
15-39 years	3,752	6.4	6.2	[6,0;6,3]	6.5	[6,3;6,7]	5.6	[5,4;5,7]
**Colorectal carcinoma**								
5-14 years	137	0.6	0.6	[0,5;0,8]	0.6	[0,5;0,8]	0.6	[0,5;0,7]
15-19 years	197	1.8	1.8	[1,6;2,1]	1.8	[1,6;2,1]	1.8	[1,6;2,1]
20-24 years	280	2.5	2.5	[2,2;2,8]	2.5	[2,2;2,8]	2.5	[2,2;2,8]
25-29 years	348	2.9	2.9	[2,6;3,2]	2.9	[2,6;3,2]	2.9	[2,6;3,2]
30-34 years	595	4.9	4.9	[4,5;5,3]	4.9	[4,5;5,3]	4.9	[4,5;5,3]
35-39 years	1,013	8.0	8.0	[7,5;8,5]	8.0	[7,5;8,5]	8.0	[7,5;8,5]
40-49 years	5,419	20.3	20.2	[19,7;20,7]	20.2	[19,7;20,7]	20.2	[19,7;20,7]
15-39 years	2,433	4.1	4.0	[3,9;4,2]	4.2	[4,0;4,4]	3.7	[3,5;3,8]
**Rest of carcinoma of gastrointestinal tract**								
5-14 years	16	0.1	0.1	[0,0;0,1]	0.1	[0,0;0,1]	0.1	[0,0;0,1]
15-19 years	24	0.2	0.2	[0,1;0,3]	0.2	[0,1;0,3]	0.2	[0,1;0,3]
20-24 years	78	0.7	0.7	[0,5;0,8]	0.7	[0,5;0,8]	0.7	[0,5;0,8]
25-29 years	186	1.6	1.6	[1,3;1,8]	1.6	[1,3;1,8]	1.6	[1,3;1,8]
30-34 years	350	2.9	2.9	[2,6;3,2]	2.9	[2,6;3,2]	2.9	[2,6;3,2]
35-39 years	681	5.4	5.4	[5,0;5,8]	5.4	[5,0;5,8]	5.4	[5,0;5,8]
40-49 years	4,321	16.2	16.1	[15,6;16,6]	16.1	[15,6;16,6]	16.1	[15,6;16,6]
15-39 years	1,319	2.2	2.1	[2,0;2,3]	2.3	[2,2;2,4]	1.9	[1,8;2,0]
**Carcinoma of lung, bronchus, and trachea**								
5-14 years	11	0.1	0.1	[0,0;0,1]	0.1	[0,0;0,1]	0.1	[0,0;0,1]
15-19 years	23	0.2	0.2	[0,1;0,3]	0.2	[0,1;0,3]	0.2	[0,1;0,3]
20-24 years	34	0.3	0.3	[0,2;0,4]	0.3	[0,2;0,4]	0.3	[0,2;0,4]
25-29 years	73	0.6	0.6	[0,5;0,8]	0.6	[0,5;0,8]	0.6	[0,5;0,8]
30-34 years	190	1.6	1.6	[1,3;1,8]	1.6	[1,3;1,8]	1.6	[1,3;1,8]
35-39 years	510	4.0	4.0	[3,7;4,4]	4.0	[3,7;4,4]	4.0	[3,7;4,4]
40-49 years	4,932	18.4	18.4	[17,8;18,9]	18.4	[17,8;18,9]	18.4	[17,8;18,9]
15-39 years	830	1.4	1.3	[1,2;1,4]	1.4	[1,3;1,5]	1.2	[1,1;1,2]
**Skin carcinoma**								
5-14 years	84	0.4	0.4	[0,3;0,5]	0.4	[0,3;0,5]	0.4	[0,3;0,5]
15-19 years	122	1.1	1.1	[0,9;1,3]	1.1	[0,9;1,3]	1.1	[0,9;1,3]
20-24 years	471	4.2	4.2	[3,8;4,5]	4.2	[3,8;4,5]	4.2	[3,8;4,5]
25-29 years	1,221	10.3	10.3	[9,7;10,8]	10.3	[9,7;10,8]	10.3	[9,7;10,8]
30-34 years	2,832	23.2	23.2	[22,3;24,1]	23.2	[22,3;24,1]	23.2	[22,3;24,1]
35-39 years	5,784	45.5	45.5	[44,3;46,7]	45.5	[44,3;46,7]	45.5	[44,3;46,7]
40-49 years	29,229	109.3	109.0	[107,7;110,2]	109.0	[107,7;110,2]	109.0	[107,7;110,2]
15-39 years	10,430	17.7	16.8	[16,5;17,2]	18.1	[17,8;18,5]	14.5	[14,2;14,8]
**Breast carcinoma**								
5-14 years	-	-	-	[0,0;0,0]	-	[0,0;0,0]	-	[0,0;0,0]
15-19 years	3	0.0	0.0	[0,0;0,1]	0.0	[0,0;0,1]	0.0	[0,0;0,1]
20-24 years	105	0.9	0.9	[0,7;1,1]	0.9	[0,7;1,1]	0.9	[0,7;1,1]
25-29 years	763	6.4	6.4	[6,0;6,9]	6.4	[6,0;6,9]	6.4	[6,0;6,9]
30-34 years	2,250	18.4	18.4	[17,7;19,2]	18.4	[17,7;19,2]	18.4	[17,7;19,2]
35-39 years	5,102	40.1	40.1	[39,0;41,2]	40.1	[39,0;41,2]	40.1	[39,0;41,2]
40-49 years	27,608	103.2	103.0	[101,7;104,2]	103.0	[101,7;104,2]	103.0	[101,7;104,2]
15-39 years	8,223	14.0	13.2	[12,9;13,5]	14.4	[14,0;14,7]	11.1	[10,8;11,3]
**Carcinoma of genital sites excluding ovary and testis**								
5-14 years	-	-	-	[0,0;0,0]	-	[0,0;0,0]	-	[0,0;0,0]
15-19 years	4	0.0	0.0	[0,0;0,1]	0.0	[0,0;0,1]	0.0	[0,0;0,1]
20-24 years	54	0.5	0.5	[0,3;0,6]	0.5	[0,3;0,6]	0.5	[0,3;0,6]
25-29 years	324	2.7	2.7	[2,4;3,0]	2.7	[2,4;3,0]	2.7	[2,4;3,0]
30-34 years	764	6.3	6.3	[5,8;6,7]	6.3	[5,8;6,7]	6.3	[5,8;6,7]

35-39 years	1,358	10.7	10.7	[10,1;11,3]	10.7	[10,1;11,3]	10.7	[10,1;11,3]
40-49 years	5,609	21.0	20.9	[20,4;21,5]	20.9	[20,4;21,5]	20.9	[20,4;21,5]
15-39 years	2,504	4.2	4.0	[3,9;4,2]	4.4	[4,2;4,5]	3.4	[3,3;3,6]
**Carcinoma of urinary tract**								
5-14 years	10	0.0	0.0	[0,0;0,1]	0.0	[0,0;0,1]	0.0	[0,0;0,1]
15-19 years	12	0.1	0.1	[0,0;0,2]	0.1	[0,0;0,2]	0.1	[0,0;0,2]
20-24 years	38	0.3	0.3	[0,2;0,4]	0.3	[0,2;0,4]	0.3	[0,2;0,4]
25-29 years	87	0.7	0.7	[0,6;0,9]	0.7	[0,6;0,9]	0.7	[0,6;0,9]
30-34 years	205	1.7	1.7	[1,4;1,9]	1.7	[1,4;1,9]	1.7	[1,4;1,9]
35-39 years	499	3.9	3.9	[3,6;4,3]	3.9	[3,6;4,3]	3.9	[3,6;4,3]
40-49 years	2,946	11.0	11.0	[10,6;11,4]	11.0	[10,6;11,4]	11.0	[10,6;11,4]
15-39 years	841	1.4	1.4	[1,3;1,4]	1.5	[1,4;1,6]	1.2	[1,1;1,2]
**Other invasive carcinomas**								
5-14 years	5	0.0	0.0	[0,0;0,0]	0.0	[0,0;0,0]	0.0	[0,0;0,0]
15-19 years	12	0.1	0.1	[0,0;0,2]	0.1	[0,0;0,2]	0.1	[0,0;0,2]
20-24 years	21	0.2	0.2	[0,1;0,3]	0.2	[0,1;0,3]	0.2	[0,1;0,3]
25-29 years	51	0.4	0.4	[0,3;0,5]	0.4	[0,3;0,5]	0.4	[0,3;0,5]
30-34 years	61	0.5	0.5	[0,4;0,6]	0.5	[0,4;0,6]	0.5	[0,4;0,6]
35-39 years	154	1.2	1.2	[1,0;1,4]	1.2	[1,0;1,4]	1.2	[1,0;1,4]
40-49 years	858	3.2	3.2	[3,0;3,4]	3.2	[3,0;3,4]	3.2	[3,0;3,4]
15-39 years	299	0.5	0.5	[0,4;0,5]	0.5	[0,5;0,6]	0.4	[0,4;0,5]
**Other neoplasms**								
5-14 years	125	0.6	0.6	[0,5;0,7]	0.6	[0,5;0,7]	0.6	[0,5;0,7]
15-19 years	27	0.3	0.3	[0,2;0,3]	0.3	[0,2;0,3]	0.3	[0,2;0,3]
20-24 years	62	0.5	0.5	[0,4;0,7]	0.5	[0,4;0,7]	0.5	[0,4;0,7]
25-29 years	69	0.6	0.6	[0,4;0,7]	0.6	[0,4;0,7]	0.6	[0,4;0,7]
30-34 years	104	0.9	0.9	[0,7;1,0]	0.9	[0,7;1,0]	0.9	[0,7;1,0]
35-39 years	176	1.4	1.4	[1,2;1,6]	1.4	[1,2;1,6]	1.4	[1,2;1,6]
40-49 years	799	3.0	3.0	[2,8;3,2]	3.0	[2,8;3,2]	3.0	[2,8;3,2]
15-39 years	438	0.7	0.7	[0,7;0,8]	0.8	[0,7;0,8]	0.7	[0,6;0,7]

Source: Belgian Cancer Registry. Abbreviations: N = number of cases, CR = crude rate (number per 100.000), WSR = world standardized rate, ESR = European standardized rate (number per 100.000), CI = confidential interval.
