# Supplementary material for: Cancer Burden in Adolescents and Young Adults in Belgium: Trends to Incidence Stabilisation in Recent Years with Improved Survival
Source: Cancers (Basel). 2025 May 1;17(9):1543. doi: 10.3390/cancers17091543 (PMC12071148; doi:10.3390/cancers17091543)
Supplement: Supplementary file 1 [file cancers-17-01543-s001.zip › Table S3 Cancer causes of death.pdf]

Table S3. Leading cancer causes of death, Belgium, 2004-2019.

5-14 years			15-29 years			30-39 years			40-49 years		
**Cancer type**	**N**	**MR**	**Cancer type**	**N**	**MR**	**Cancer type**	**N**	**MR**	**Cancer type**	**N**	**MR**
All sites*	438	2.2 [[1,4;3,0]	All sites*	1,310	4.1 [[3,3;5,0]	All sites*	3,450	14.8 [[12,9;16,9]	All sites*	13,959	55.0 [[51,4;58,6]
CNS tumors	207	1.0 [0,5;1,6]	Hematological malignancies	367	1.1 [0,7;1,6]	Breast carcinoma	601	2.6 [1,8;3,5]	Lung, bronchus, trachea carcinoma	2948	11.6 [10,1;13,6]
Hematological malignancies	128	0.6 [0,2;1,0]	CNS tumors	244	0.8 [0,4;1,2]	CNS tumors	410	1.8 [1,1;2,5]	Breast carcinoma	2314	9.1 [7,6;10,6]
Sarcoma	64	0.3 [0,0;0,6]	Sarcoma	235	0.7 [0,4;1,1]	Hematological malignancies	398	1.7 [1,0;2,3]	Carcinoma of GI tract, except colorectal and pancreas	1466	5.8 [4,6;7,0]
Other invasive carincoma	18	0.1 [-0,1;0,2]	Carcinoma of GI tract, except colorectal and pancreas	61	0.2 [0,0;0,5]	Lung, bronchus, trachea carcinoma	347	1.5 [0,9;2,1]	Colorectal carcinoma	960	3.8 [2,9;4,8]
Carcinoma of the urinary tract	10	0.0 [-0,1;0,2]	Skin melanoma	52	0.2 [0,0;0,4]	Carcinoma of GI tract, except colorectal and pancreas	334	1.4 [0,8;2,0]	CNS tumors	927	3.7 [2,7;4,6]

Source: Statbel. Abbreviations: MR = European (ESP 2013) standardized mortality rate (number per 100,000)

* All invasive tumours except non-melanoma of the skin
