# Supplementary material for: Cancer Burden in Adolescents and Young Adults in Belgium: Trends to Incidence Stabilisation in Recent Years with Improved Survival
Source: Cancers (Basel). 2025 May 1;17(9):1543. doi: 10.3390/cancers17091543 (PMC12071148; doi:10.3390/cancers17091543)
Supplement: Supplementary file 1 [file cancers-17-01543-s001.zip › Table S4 AAPC.pdf]

Table S4. (A)APC estimates of incidence (APC I) for period 2004-2020 and of mortality (APC M) for period 2004-2019, by age category and by sex, Belgium.

Age group (years)	Incidence			Mortality		
	APC I [95% CI]		AAPC I [95% CI]	APC M [95% CI]		AAPC M [95% CI]
	Year		2004-2020	Year		2004-2019
**All**						
5-14	-	-	-0.13 [-0.60;0.34]	-	-	-1.35 [-4.21;0.97]
15-29	2004-2015<br>2015-2020	0.31* [0.21;0.46]<br>-0.63* [-1.12;-0.32]	0.02 [-0.07;0.10]	-	-	-1.86* [-3.02;-0.92]
30-39	-	-	0.20* [0.06;0.34]	-	-	-0.82* [-1.27;-0.43]
40-49	-	-	-0.04 [-0.08;0.01]	-	-	-0.86* [-1.01;-0.73]
15-39	2004-2015<br>2015-2020	0.45* [0.30;0.98]<br>-0,38 [-1.68;0.10]	0.19* [0.02;0.36]	-	-	-1.16* [-1.72;-0.69]
**Males**						
5-14	-	-	-0.18 [--0.17;0.70]	-	-	0.40 [-2.53;3.59]
15-29	-	-	0.12 [-0.06;0.30]	-	-	-1.59* [-3.05;-0.41]
30-39	-	-	0.34* [0.15;0.56]	-	-	-0.72* [-1.20;-0.28]
40-49	-	-	-0.09 [-0.23;0.05]	-	-	-1.10* [-1.32;-0.92]
15-39	-	-	0.27* [0.15;0.40]	-	-	-0.88* [-1.72;-0.17]
**Females**						
5-14	-	-	-0.05 [--0.05;-0.69]	-	-	-6.23 [-0.87 – 0.75]
15-29	2004-2015<br>2015-2020	0,32* [0.13;1.38]<br>-0,78* [-2.84;-0.11]	-0.02 [-0.29; 0.27]	-	-	-2.16* [-4.30;-0.57]
30-39	2004-2010<br>2010-2020	0.45* [0.21;1.34]<br>0.02 [-0.45;0.12]	0.18* [0.08;0.29]	-	-	-1.04* [-1.43;-0.64]
40-49	-	-	0.00 [0.00;0.00]	2004-2007<br>2007-2020	0.20 [-0.56;1.63]<br>-0.78* [-1.48;-0.67]	-0.58* [-0.79;-0.45]
15-39	-	-	0.14* [0.01;0.28]	-	-	-1.13* [-1.59;-0.74]
**Hematological malignancies**						
5-14				-	-	-
15-29				-	-	-
30-39				-	-	-1.80 [-6.91;1.93]
40-49				-	-	-2.01* [-3.69;-0.70]
15-39				-	-	-
**Hodgkin lymphomas (HL)**						
5-14	-	-	-			
15-29	-	-	0.59* [0.05;1.16]			
30-39	-	-	2.04* [0.78;3.74]			
40-49	-	-	0.12 [-1.43;1.74]			
15-39	-	-	1.07* [0.41;1.85]			
**Mature B-cell neoplasms**						
5-14	-	-	-			
15-29	-	-	0.45 [-2.97;4.18]			
30-39	-	-	0.12 [-0.79;1.09]			
40-49	-	-	-0.19 [-0.65;0.25]			
15-39	-	-	0.22 [-0.59;1.09]			
**Mature T-cell and NK-cell neoplasms**						
5-14	-	-	-			
15-29	-	-	-			
30-39	-	-	-			
40-49	-	-	4.92* [2.55;9.73]			
15-39	-	-	-			
**Other lymphoid neoplasms**						
5-14	-	-	-			
15-29	-	-	-			
30-39	-	-	-			

40-49	-	-	-			
15-39	-	-	-			
**Precursor hematopoietic neoplasms**						
5-14	-	-	0.22 [-1.10;1.68]			
15-29	-	-	1.66 [-0.55;4.72]			
30-39	-	-	-1.41 [-3.20;0.09]			
40-49	-	-	-0.37 [-2.12;1.35]			
15-39	-	-	0.04 [-1.57;1.77]			
**Chronic myeloid neoplasms**						
5-14	-	-	-			
15-29	-	-	-			
30-39	-	-	2.43* [1,33;4,03]			
40-49	-	-	1.49* [0,86;2,27]			
15-39	-	-	3.13* [1,21;6,00]			
**Histiocytic and dendritic cell neoplasms (HDCN)**						
5-14	-	-	-			
15-29	-	-	-			
30-39	-	-	-			
40-49	-	-	-			
15-39	-	-	-			
**Central nervous system (CNS)**						
5-14	-	-	-1.26 [-1.19;4.84]	-	-	-
15-29	-	-	-1.73 [-4.17;-0.30]	-	-	-
30-39	-	-	0.56 [-0.63;1.90]	-	-	-0.37 [-4.86;3.15]
40-49	2004-2013<br>2013-2020	-1.27* [-6.22;-0.02]<br>1.38 [-0.33;7.45]	-0.12 [-0.79;0.50]	-	-	-0.87 [-2.42;0.45]
15-39	-	-	-0.33 [-1.50;0.84]	-	-	-
**Sarcoma**						
5-14	-	-	-1.36 [-4.69;1.50]	-	-	-
15-29	-	-	-1.41* [-2.64;-0.38]	-	-	-
30-39	-	-	0.07 [-0.75;0.91]	-	-	-
40-49	-	-	0.03 [-0.57;0.66]	-	-	-
15-39	-	-	-0.54 [-1.23;0.13]	-	-	-
**Skin melanoma**						
5-14	-	-	-	-	-	-
15-29	2004-2007<br>2007-2010<br>2010-2020	-5.14 [-12.73;1.06]<br>8.32* [1.72;12.45]<br>-1.60* [-2.88;-0.73]	-0.50 [-1.13;0.27]	-	-	-
30-39	-	-	0.69* [0.24;1.22]	-	-	-
40-49	2004-2014<br>2014-2020	1.23* [1.76;4.76]<br>0.38 [-2.29;1.24]	1.53* [1.14;2.12]	2005-2008<br>2008-2019	17.89 [-2.60;101.68]<br>-4.55* [-67.23;-.,50]	-0.13 [-16.09;6.39]
15-39	2004-2010<br>2010-2020	1.99* [0.8;9.0]<br>-0.02 [-3.1;0.5]	0.73* [0.2;1.3]	-	-	-
**Testis**						
5-14	-	-	-	-	-	-
15-29	-	-	-	-	-	-
30-39	2004-2015<br>2015-2020	2.51* [1.70;19.57]<br>-0.47 [-8.27;1.66]	1.57* [0,65;3,58]	-	-	-
40-49	-	-	3.24* [2.26;4.68]	-	-	-
15-39	-	-	1.11* [0.68;1.62]	-	-	-
**Gonadal, non-testicular tumors**						
5-14	-	-	-	-	-	-
15-29	-	-	-	-	-	-
30-39	-	-	-0.52 [-3.68;2.31]	-	-	-
40-49	-	-	-2.07* [-3.27;-1.09]	2004-2007<br>2007-2017<br>2017-2020	45.11* [35.59;62.02]<br>-19.39* [-27.00;-18.05]<br>82.41* [12.12;132.93]	1.10 [-2.21;3.40]

15-39	-	-	-1.67 [-6.89;2.94]	-	-	-
**Thyroid carcinoma**						
5-14	-	-	-	-	-	-
15-29	2004-2018<br>2018-2020	2.49* [1.55;6.54]<br>-16.74* [-28.47;-0.29]	-0.13 [-1.80;2.50]	-	-	-
30-39	2004-2011<br>2011-2020	3.86* [2.41;7.22]<br>-0.77 [-2.24;0.11]	1.23* [0.66;2.04]	-	-	-
40-49	2004-2015<br>2015-2020	1.64* [1.08;5.55]<br>-1.28 [-7.27;0.48]	0.72* [0.00;1.59]	-	-	-
15-39	2004-2012<br>2012-2020	3.99* [2.64;6.71]<br>-1.30* [-3.17;-0.18]	1.31* [0.67;2.13]	-	-	-
**Other carcinoma of head and neck**						
5-14	-	-	-	-	-	-
15-29	-	-	-	-	-	-
30-39	-	-	-0.44 [-2.42;-1.43]	-	-	-
40-49	-	-	-2.00* [-2.40;-1.69]	-	-	-8.42* [-11.52;7.28]
15-39	-	-	-2.35 [-5.81;0.49]	-	-	-
**Colorectal carcinomas**						
5-14	-	-	-	-	-	-
15-29	-	-	1.19 [-0.91;3.86]	-	-	-
30-39	-	-	1.54* [1.00;2.20]	-	-	-
40-49	-	-	-0.16 [-0.40;0.07]	-	-	-1.15 [-2.77;0.17]
15-39	2004-2013<br>2013-2020	3.40* [2.11;11.14]<br>-0.36 [-4.96;1.17]	1.74* [0.92;3.03]	-	-	-
**Carcinoma of pancreas**						
5-14	-	-	-	-	-	-
15-29	-	-	-	-	-	-
30-39	-	-	-	-	-	-
40-49	-	-	1.45* [0.23;2.98]	-	-	-0.93 [-3.00;0.86]
15-39	-	-	-	-	-	-
**Rest of carcinomas GI tract**						
5-14	-	-	-	-	-	-
15-29	-	-	-	-	-	-
30-39	-	-	0.68 [-0.87;2.54]	-	-	0.80 [-3.48;5.14]
40-49	-	-	-0.12 [-0.54;0.31]	-	-	-0.49 [-1.14;0.10]
15-39	-	-	0.99 [-1.15;3.52]	-	-	-
**Carcinoma of lung, bronchus, and trachea**						
5-14	-	-	-	-	-	-
15-29	-	-	-	-	-	-
30-39	-	-	0.09 [-1.28;1.54]	-	-	-1,61 [-5,86;1,86]
40-49	2004-2007<br>2007-2013<br>2013-2020	0.88 [-0.23;3.11]<br>-2.01* [-3.39;-1.52]<br>-0.42 [-0.95;1.33]	-0.78* [-0.97;-0.55]	2004-2009<br>2009-2019	-0.68 [-1.97;4.32]<br>-3.04* [-7.77;-2.51]	-2.26* [-3.35;-1.69]
15-39	-	-	0.39 [-3.03;4.63]	-	-	-
**Carcinoma of breast**						
5-14	-	-	-	-	-	-
15-29	-	-	1.49* [0.00;3.44]	-	-	-
30-39	-	-	0.20 [0.00;0.42]	-	-	-3.22* [-7.21;-0.61]
40-49	-	-	-0.14* [-0.22;-0.08]	-	-	-1.51* [-2.10;-1.06]
15-39	-	-	0.29* [0.06;0.53]	-	-	-
**Carcinoma of genital sites excl. ovary and testis**						
5-14	-	-	-	-	-	-
15-29	-	-	-	-	-	-
30-39	-	-	-0.61* [-1.01;-0.23]	-	-	-
40-49	-	-	-0.44* [-0.79;-0.11]	-	-	-2.75* [-4.57;-1.42]
15-39	-	-	-0.89* [-1.65;-0.15]	-	-	-
**Carcinoma of urinary tract**						

5-14	-	-	-	-	-	-
15-29	-	-	-	-	-	-
30-39	2004-2016<br>2016-2020	2.21* [0.66;9.74]<br>-12.51* [-38.25;-1.91]	-1.69 [-5.98;1.51]	-	-	-
40-49	-	-	0.18 [-0.27;0.64]	-	-	-3.87* [-7.98;-1.08]
15-39	-	-	2.17 [-1.41;6.92]	-	-	-
**Other invasive tumors**						
5-14	-	-	-	-	-	-
15-29	-	-	-	-	-	-
30-39	-	-	-	-	-	-
40-49	-	-	-	-	-	-3.91* [-6.14;-2.57]
15-39	-	-	-	-	-	-
**Other invasive carcinomas**						
5-14	-	-	-			
15-29	-	-	-			
30-39	-	-	-			
40-49	-	-	-2.32* [-3.90;-1.20]			
15-39	-	-	-			
**Other neoplasms**						
5-14	-	-	-			
15-29	-	-	-			
30-39	-	-	-			
40-49	-	-	-0.49 [-2.64;1.63]			
15-39	-	-	-			

Source: Belgian Cancer Registry. Abbreviations: APC = annual percentual change, CI = confidential interval. * Indicates that the (A)APC is significantly different from zero at alpha = 0.05 level, - Indicates that the Joinpoint regression program did not select/withhold 1 or more joinpoints. ASR=ESR2013
