# Supplementary material for: Cancer Burden in Adolescents and Young Adults in Belgium: Trends to Incidence Stabilisation in Recent Years with Improved Survival
Source: Cancers (Basel). 2025 May 1;17(9):1543. doi: 10.3390/cancers17091543 (PMC12071148; doi:10.3390/cancers17091543)
Supplement: Supplementary file 1 [file cancers-17-01543-s001.zip › Table S5 Survival.pdf]

Table S5. Relative survival by sex, tumour type and age category, Belgium, 2004-2020.

Age group (years)	N at risk	Relative Survival (%)					
		1 year	95% CI	5 year	95% CI	10 year	95% CI
**Males**							
5-14	1,470	93.5%	[92.2%:94.7%]	82.9%	[80.8%:84.8%]	79.8%	[77.4%:81.9%]
15-19	1,374	94.3%	[92.9%:95.4%]	85.5%	[83.4%:87.3%]	83.9%	[81.7%:85.9%]
20-24	2,105	96.2%	[95.3%:96.9%]	88.8%	[87.3%:90.2%]	86.2%	[84.4%:87.7%]
25-29	3,119	95.8%	[95.1%:96.5%]	88.9%	[87.6%:90.0%]	86.2%	[84.8%:87.6%]
30-34	4,090	94.1%	[93.3%:94.8%]	85.0%	[83.8%:86.1%]	81.7%	[80.3%:83.1%]
35-39	5,540	90.9%	[90.1%:91.6%]	79.3%	[78.2%:80.5%]	74.9%	[73.6%:76.2%]
40-49	25,249	85.1%	[84.6%:85.5%]	67.7%	[67.1%:68.3%]	62.3%	[61.6%:63.0%]
15-39	16,075	93.7%	[93.3%:94.0%]	84.4%	[83.8%:85.0%]	81.1%	[80.4%:81.8%]
**Females**							
5-14	1,177	94.2%	[92.7%:95.4%]	84.9%	[82.7%:86.9%]	83.5%	[81.1%:85.6%]
15-19	1,156	96.2%	[95.0%:97.2%]	89.2%	[87.2%:90.9%]	87.3%	[85.1%:89.2%]
20-24	2,143	97.0%	[96.2%:97.6%]	91.8%	[90.6%:93.0%]	89.7%	[88.2%:91.1%]
25-29	4,002	96.9%	[96.4%:97.4%]	90.3%	[89.3%:91.2%]	87.7%	[86.5%:88.8%]
30-34	6,991	96.7%	[96.2%:97.1%]	88.5%	[87.7%:89.2%]	84.6%	[83.5%:85.5%]
35-39	12,137	96.6%	[96.3%:96.9%]	87.9%	[87.3%:88.5%]	83.7%	[82.9%:84.5%]
40-49	51,753	95.0%	[94.8%:95.2%]	85.9%	[85.6%:86.2%]	81.5%	[81.1%:81.9%]
15-39	26,244	96.7%	[96.5%:96.9%]	88.9%	[88.5%:89.3%]	85.3%	[84.8%:85.7%]
**Hodgkin Lymphoma**							
5-14	235	99.2%	[96.7%:99.9%]	98.2%	[94.9%:99.5%]	235	99.2%
15-19	501	97.2%	[95.1%:98.4%]	96.6%	[94.2%:98.1%]	501	97.2%
20-24	622	98.7%	[97.2%:99.4%]	97.5%	[95.4%:98.8%]	622	98.7%
25-29	569	97.7%	[95.9%:98.8%]	97.3%	[95.1%:98.6%]	569	97.7%
30-34	467	96.4%	[94.0%:97.9%]	96.6%	[94.0%:98.2%]	467	96.4%
35-39	428	96.7%	[94.2%:98.3%]	95.1%	[91.7%:97.4%]	428	96.7%
40-49	669	94.3%	[92.0%:96.0%]	91.7%	[88.5%:94.2%]	669	94.3%
15-39	2,587	97.4%	[96.7%:98.1%]	96.7%	[95.7%:97.5%]	2,587	97.4%
**Mature B-cell neoplasms**							
5-14	183	94.5%	[89.9%:97.0%]	94.6%	[90.1%:97.2%]	183	94.5%
15-19	151	89.9%	[83.6%:93.8%]	88.9%	[82.1%:93.3%]	151	89.9%
20-24	203	86.2%	[80.6%:90.3%]	85.8%	[80.0%:90.1%]	203	86.2%
25-29	284	89.3%	[84.9%:92.5%]	88.4%	[83.5%:91.9%]	284	89.3%
30-34	458	89.0%	[85.7%:91.7%]	87.9%	[84.2%:90.9%]	458	89.0%
35-39	725	89.5%	[86.9%:91.6%]	86.0%	[82.8%:88.8%]	725	89.5%
40-49	3,541	88.6%	[87.4%:89.7%]	82.9%	[81.3%:84.4%]	3,541	88.6%
15-39	1,820	89.1%	[87.5%:90.5%]	87.2%	[85.3%:88.8%]	1,820	89.1%
**Mature T- and NK-cell neoplasms**							
5-14	46					46	
15-19	43					43	
5-19	58	80.9%	[67.9%:89.0%]	77.6%	[62.9%:87.2%]	58	80.9%
20-24	85	90.8%	[82.1%:95.4%]	91.1%	[82.5%:95.8%]	85	90.8%
25-29	127	84.9%	[77.2%:90.2%]	83.2%	[74.9%:89.1%]	127	84.9%
30-34	132	84.1%	[76.1%:89.6%]	80.8%	[71.6%:87.4%]	132	84.1%
35-39	406	79.0%	[74.4%:82.9%]	75.1%	[69.6%:79.8%]	406	79.0%
40-49	445	86.3%	[82.6%:89.3%]	84.5%	[80.4%:87.9%]	445	86.3%
15-39	46					46	

**Other lymphoid neoplasms**							
5-14	8					8	
15-19	16					16	
5-19	22					22	
20-24	24					24	
25-29	20					20	
30-34	28					28	
35-39	110	84.8%	[76.1%:90.7%]	78.5%	[68.1%:86.2%]	110	84.8%
40-49	110	93.0%	[86.2%:96.6%]	93.5%	[86.6%:97.1%]	110	93.0%
15-39	8					8	
**Precursor hematopoietic neoplasms**							
5-14	665	87.1%	[84.3%:89.5%]	85.3%	[82.1%:87.9%]	665	87.1%
15-19	285	72.5%	[66.8%:77.5%]	70.6%	[64.6%:75.7%]	285	72.5%
20-24	224	66.8%	[60.0%:72.8%]	62.2%	[54.7%:68.8%]	224	66.8%
25-29	210	64.4%	[57.1%:70.8%]	60.2%	[52.1%:67.4%]	210	64.4%
30-34	268	58.0%	[51.6%:63.8%]	52.7%	[46.0%:59.0%]	268	58.0%
35-39	295	57.8%	[51.7%:63.4%]	54.6%	[48.2%:60.5%]	295	57.8%
40-49	846	54.4%	[50.9%:57.9%]	50.7%	[47.0%:54.4%]	846	54.4%
15-39	1,279	63.8%	[61.0%:66.5%]	60.1%	[57.1%:62.9%]	1,279	63.8%
**Chronic myeloid neoplasms**							
5-14	102	88.0%	[79.8%:93.1%]	86.8%	[78.2%:92.3%]	102	88.0%
15-19	81	89.9%	[80.6%:94.9%]	90.1%	[80.8%:95.1%]	81	89.9%
20-24	130	96.7%	[91.2%:98.9%]	93.0%	[85.0%:96.9%]	130	96.7%
25-29	207	94.5%	[90.0%:97.1%]	93.9%	[88.9%:96.9%]	207	94.5%
30-34	295	93.7%	[89.9%:96.1%]	90.8%	[86.0%:94.1%]	295	93.7%
35-39	428	94.7%	[91.9%:96.7%]	90.8%	[86.5%:93.8%]	428	94.7%
40-49	1,545	91.6%	[90.0%:93.1%]	88.0%	[85.7%:90.1%]	1,545	91.6%
15-39	1,140	94.3%	[92.7%:95.6%]	91.6%	[89.3%:93.4%]	1,140	94.3%
**Histiocytic and dendritic neoplasms**							
5-14	96	98.0%	[92.0%:99.6%]	98.1%	[92.1%:99.7%]	96	98.0%
15-19	27					27	
5-19	21					21	
20-24	23					23	
25-29	22					22	
30-34	42					42	
35-39	67	86.1%	[74.1%:93.0%]	85.2%	[72.3%:92.9%]	67	86.1%
40-49	135	94.2%	[88.4%:97.2%]	90.5%	[81.1%:95.6%]	135	94.2%
15-39	96	98.0%	[92.0%:99.6%]	98.1%	[92.1%:99.7%]	96	98.0%
**CNS**							
5-14	479	62.9%	[58.3%:67.2%]	58.6%	[53.7%:63.1%]	479	62.9%
15-19	198	74.1%	[67.2%:79.8%]	66.3%	[58.4%:73.1%]	198	74.1%
20-24	269	68.7%	[62.5%:74.1%]	57.3%	[50.3%:63.6%]	269	68.7%
25-29	399	70.2%	[65.2%:74.7%]	57.6%	[51.8%:63.0%]	399	70.2%
30-34	481	67.1%	[62.4%:71.3%]	50.9%	[45.4%:56.2%]	481	67.1%
35-39	556	62.4%	[58.0%:66.5%]	47.8%	[42.7%:52.7%]	556	62.4%
40-49	1,743	43.2%	[40.7%:45.6%]	34.4%	[31.8%:37.0%]	1,743	43.2%
15-39	1,902	67.3%	[65.1%:69.5%]	54.0%	[51.3%:56.6%]	1,902	67.3%
**Sarcoma**							
5-14	416	77.1%	[72.6%:81.0%]	73.9%	[69.0%:78.2%]	416	77.1%
15-19	377	70.2%	[65.1%:74.7%]	67.5%	[62.1%:72.2%]	377	70.2%
20-24	310	77.7%	[72.4%:82.2%]	75.2%	[69.6%:80.0%]	310	77.7%

25-29	395	83.1%	[78.8%:86.6%]	76.8%	[71.6%:81.2%]	395	83.1%
30-34	496	81.1%	[77.2%:84.4%]	76.5%	[72.0%:80.4%]	496	81.1%
35-39	667	82.3%	[79.0%:85.1%]	78.5%	[74.8%:81.8%]	667	82.3%
40-49	2,087	82.1%	[80.3%:83.8%]	76.2%	[74.0%:78.3%]	2,087	82.1%
15-39	2,241	79.5%	[77.7%:81.2%]	75.5%	[73.5%:77.4%]	2,241	79.5%
**Skin melanoma**							
5-14	46					46	
15-19	165	97.7%	[93.7%:99.2%]	96.0%	[90.8%:98.4%]	165	97.7%
5-19	615	97.4%	[95.7%:98.5%]	96.4%	[94.2%:97.8%]	615	97.4%
20-24	1,166	96.7%	[95.4%:97.7%]	95.3%	[93.6%:96.6%]	1,166	96.7%
25-29	1,696	95.5%	[94.3%:96.5%]	93.8%	[92.2%:95.0%]	1,696	95.5%
30-34	2,424	95.2%	[94.2%:96.1%]	93.3%	[92.0%:94.5%]	2,424	95.2%
35-39	7,046	94.5%	[93.9%:95.1%]	92.6%	[91.7%:93.4%]	7,046	94.5%
40-49	6,031	95.9%	[95.3%:96.4%]	94.2%	[93.4%:94.9%]	6,031	95.9%
15-39	46					46	
**All gonadal and related cancers**							
5-14	95	94.5%	[87.3%:97.7%]	93.2%	[85.2%:97.0%]	95	94.5%
15-19	280	96.2%	[93.1%:98.0%]	96.0%	[92.6%:97.9%]	280	96.2%
20-24	748	97.4%	[95.8%:98.4%]	97.1%	[95.3%:98.3%]	748	97.4%
25-29	1,248	96.3%	[95.0%:97.3%]	95.5%	[94.0%:96.7%]	1,248	96.3%
30-34	1,348	95.6%	[94.3%:96.7%]	94.7%	[93.0%:96.0%]	1,348	95.6%
35-39	1,218	91.1%	[89.2%:92.7%]	88.7%	[86.4%:90.7%]	1,218	91.1%
40-49	2,274	81.5%	[79.7%:83.2%]	73.7%	[71.4%:75.9%]	2,274	81.5%
15-39	4,795	95.0%	[94.3%:95.6%]	93.9%	[93.0%:94.6%]	4,795	95.0%
**Testis**							
5-14	4					4	
15-19	194	98.7%	[95.5%:99.8%]	99.0%	[95.8%:100.1%]	194	98.7%
20-24	628	98.8%	[97.4%:99.6%]	98.9%	[97.3%:99.8%]	628	98.8%
25-29	1,063	97.9%	[96.7%:98.7%]	97.6%	[96.2%:98.6%]	1,063	97.9%
30-34	1,164	97.9%	[96.8%:98.7%]	97.6%	[96.2%:98.7%]	1,164	97.9%
35-39	929	97.5%	[96.0%:98.5%]	97.2%	[95.4%:98.5%]	929	97.5%
40-49	1,100	97.8%	[96.5%:98.8%]	96.7%	[94.5%:98.3%]	1,100	97.8%
15-39	3,931	98.0%	[97.5%:98.5%]	97.8%	[97.1%:98.4%]	3,931	98.0%
**Ovary**							
5-14	45					45	
15-19	50	89.5%	[76.3%:95.5%]	86.8%	[72.6%:94.0%]	50	89.5%
20-24	72	88.5%	[78.1%:94.2%]	86.8%	[75.9%:93.1%]	72	88.5%
25-29	122	84.8%	[76.9%:90.2%]	80.6%	[71.7%:87.0%]	122	84.8%
30-34	126	79.8%	[71.3%:86.1%]	73.1%	[63.4%:80.7%]	126	79.8%
35-39	240	69.2%	[62.6%:74.9%]	59.3%	[52.1%:65.9%]	240	69.2%
40-49	1,111	66.3%	[63.3%:69.2%]	53.1%	[49.7%:56.5%]	1,111	66.3%
15-39	610	78.4%	[74.8%:81.6%]	71.8%	[67.7%:75.6%]	610	78.4%
**Germ cell and trophoblastic - CNS**							
5-14	42					42	
15-19	24					24	
5-19	24					24	
20-24	16					16	
25-29	9					9	
30-34	5					5	
35-39	13					13	
40-49	78	92.4%	[83.5%:96.7%]	89.1%	[78.7%:94.8%]	78	92.4%

15-39	42					42	
**Germ cell and trophoblastic excluding CNS, ovary, testis**							
5-14	4					4	
15-19	11					11	
5-19	24					24	
20-24	48					48	
25-29	49					49	
30-34	43					43	
35-39	45					45	
40-49	175	86.5%	[80.3%:91.0%]	86.9%	[80.6%:91.4%]	175	86.5%
15-39	4					4	
**All carcinomas**							
5-14	343	95.3%	[92.3%:97.2%]	93.9%	[90.3%:96.3%]	343	95.3%
15-19	569	94.0%	[91.6%:95.8%]	93.0%	[90.3%:94.9%]	569	94.0%
20-24	1,512	93.6%	[92.1%:94.7%]	91.7%	[89.9%:93.1%]	1,512	93.6%
25-29	3,703	91.1%	[90.1%:92.0%]	89.2%	[88.0%:90.3%]	3,703	91.1%
30-34	8,114	89.8%	[89.1%:90.5%]	86.8%	[85.9%:87.6%]	8,114	89.8%
35-39	16,125	88.7%	[88.2%:89.2%]	85.4%	[84.7%:86.0%]	16,125	88.7%
40-49	82,195	85.2%	[84.9%:85.4%]	81.7%	[81.3%:82.0%]	82,195	85.2%
15-39	29,776	89.7%	[89.3%:90.0%]	86.7%	[86.2%:87.2%]	29,776	89.7%
**Thyroid carcinoma**							
5-14	63	100.1%	[100.1%:100.1%]	97.6%	[83.2%:99.8%]	63	100.1%
15-19	149	99.4%	[95.1%:100.0%]	99.6%	[95.2%:100.2%]	149	99.4%
20-24	389	99.9%	[98.3%:100.1%]	100.1%	[98.5%:100.3%]	389	99.9%
25-29	657	99.2%	[98.0%:99.8%]	98.5%	[96.7%:99.5%]	657	99.2%
30-34	924	99.9%	[99.2%:100.2%]	100.0%	[98.9%:100.5%]	924	99.9%
35-39	1,274	98.7%	[97.7%:99.3%]	98.2%	[96.8%:99.1%]	1,274	98.7%
40-49	3,133	99.0%	[98.4%:99.5%]	98.3%	[97.3%:99.1%]	3,133	99.0%
15-39	3,393	99.3%	[98.9%:99.6%]	99.0%	[98.4%:99.5%]	3,393	99.3%
**Other carcinoma of head and neck**							
5-14	32					32	
15-19	38					38	
5-19	83	85.0%	[74.9%:91.3%]	80.0%	[68.6%:87.8%]	83	85.0%
20-24	124	84.7%	[76.6%:90.2%]	79.6%	[70.0%:86.5%]	124	84.7%
25-29	220	82.9%	[76.9%:87.5%]	78.7%	[71.8%:84.2%]	220	82.9%
30-34	365	74.6%	[69.6%:78.9%]	67.9%	[62.2%:73.0%]	365	74.6%
35-39	3,437	59.6%	[57.8%:61.3%]	47.1%	[45.2%:49.0%]	3,437	59.6%
40-49	826	79.9%	[76.8%:82.6%]	74.3%	[70.7%:77.5%]	826	79.9%
15-39	32					32	
**Carcinoma of GI tract**							
5-14	152	94.9%	[89.4%:97.6%]	94.0%	[88.1%:97.1%]	152	94.9%
15-19	220	94.4%	[90.3%:96.9%]	94.0%	[89.7%:96.7%]	220	94.4%
20-24	354	86.1%	[81.9%:89.4%]	83.3%	[78.5%:87.2%]	354	86.1%
25-29	516	74.6%	[70.4%:78.3%]	71.8%	[67.3%:75.9%]	516	74.6%
30-34	927	67.3%	[64.0%:70.4%]	63.8%	[60.2%:67.1%]	927	67.3%
35-39	1,657	64.1%	[61.7%:66.5%]	58.8%	[56.1%:61.4%]	1,657	64.1%
40-49	9,552	58.1%	[57.0%:59.1%]	52.3%	[51.2%:53.4%]	9,552	58.1%
15-39	3,665	70.5%	[69.0%:72.1%]	66.6%	[64.8%:68.2%]	3,665	70.5%
**Colorectal carcinomas**							
5-14	136	96.7%	[91.3%:98.8%]	96.8%	[91.4%:99.0%]	136	96.7%
15-19	196	98.0%	[94.6%:99.4%]	98.2%	[94.8%:99.6%]	196	98.0%

20-24	280	92.7%	[88.8%:95.3%]	90.4%	[85.6%:93.7%]	280	92.7%
25-29	334	84.9%	[80.3%:88.5%]	83.7%	[78.8%:87.6%]	334	84.9%
30-34	582	79.5%	[75.7%:82.8%]	77.0%	[72.9%:80.6%]	582	79.5%
35-39	995	75.7%	[72.7%:78.4%]	69.2%	[65.8%:72.4%]	995	75.7%
40-49	5,312	73.8%	[72.5%:75.1%]	67.8%	[66.3%:69.2%]	5,312	73.8%
15-39	2,381	82.1%	[80.4%:83.6%]	78.3%	[76.4%:80.1%]	2,381	82.1%
**Rest of the carcinoma of the gastrointestinal tract**							
5-14	16					16	
15-19	24					24	
20-24	75	60.3%	[47.7%:70.7%]	55.9%	[42.8%:67.2%]	75	60.3%
25-29	183	54.9%	[47.0%:62.1%]	49.8%	[41.5%:57.5%]	183	54.9%
30-34	346	46.2%	[40.6%:51.6%]	40.9%	[35.1%:46.7%]	346	46.2%
35-39	665	46.8%	[42.8%:50.6%]	42.9%	[38.8%:47.0%]	665	46.8%
40-49	4,265	38.2%	[36.7%:39.7%]	32.7%	[31.1%:34.3%]	4,265	38.2%
15-39	1,290	48.9%	[46.0%:51.7%]	44.4%	[41.4%:47.4%]	1,290	48.9%
**Carcinoma of skin**							
5-14	70	100.1%	[100.1%:100.1%]	100.2%	[100.2%:100.2%]	70	100.1%
15-19	109	97.0%	[90.7%:99.1%]	95.7%	[88.2%:98.6%]	109	97.0%
20-24	442	100.0%	[98.4%:100.2%]	99.9%	[98.1%:100.3%]	442	100.0%
25-29	1,134	99.5%	[98.7%:99.9%]	99.2%	[98.0%:99.9%]	1,134	99.5%
30-34	2,638	99.8%	[99.3%:100.0%]	99.6%	[99.0%:100.1%]	2,638	99.8%
35-39	5,369	99.7%	[99.4%:99.9%]	99.5%	[99.0%:99.9%]	5,369	99.7%
40-49	25,917	99.7%	[99.5%:99.8%]	99.8%	[99.5%:100.0%]	25,917	99.7%
15-39	9,556	99.7%	[99.5%:99.9%]	99.5%	[99.2%:99.8%]	9,556	99.7%
**Carcinoma of lung, bronchus and trachea**							
5-14	11					11	
15-19	23					23	
5-19	34					34	
20-24	72	66.7%	[54.0%:76.6%]	66.9%	[54.2%:76.9%]	72	66.7%
25-29	188	43.4%	[36.0%:50.7%]	42.8%	[35.2%:50.1%]	188	43.4%
30-34	506	32.4%	[28.2%:36.7%]	28.6%	[24.4%:33.1%]	506	32.4%
35-39	4,874	26.6%	[25.4%:28.0%]	21.5%	[20.2%:22.8%]	4,874	26.6%
40-49	823	41.8%	[38.2%:45.3%]	39.3%	[35.7%:42.9%]	823	41.8%
15-39	11					11	
**Carcinoma of breast**							
5-14	-	-	-	-	-	-	-
15-19	2					2	
5-19	105	86.9%	[78.3%:92.2%]	78.2%	[67.4%:85.8%]	105	86.9%
20-24	759	88.5%	[85.8%:90.8%]	84.1%	[80.7%:87.0%]	759	88.5%
25-29	2,242	88.6%	[87.1%:89.9%]	81.1%	[79.0%:83.0%]	2,242	88.6%
30-34	5,084	90.8%	[89.9%:91.6%]	84.7%	[83.5%:85.9%]	5,084	90.8%
35-39	27,534	94.1%	[93.7%:94.4%]	89.9%	[89.4%:90.3%]	27,534	94.1%
40-49	8,192	89.9%	[89.2%:90.6%]	83.6%	[82.6%:84.5%]	8,192	89.9%
15-39	-	-	-	-	-	-	-
**Carcinoma of genital sites excluding ovary and testis**							
5-14	-	-	-	-	-	-	-
15-19	4					4	
5-19	53	86.2%	[72.9%:93.2%]	82.7%	[67.3%:91.3%]	53	86.2%
20-24	324	89.7%	[85.7%:92.6%]	87.9%	[83.4%:91.3%]	324	89.7%
25-29	755	88.1%	[85.5%:90.3%]	86.9%	[84.0%:89.3%]	755	88.1%
30-34	1,341	87.3%	[85.3%:89.1%]	85.2%	[83.0%:87.2%]	1,341	87.3%

35-39	5,550	86.4%	[85.4%:87.4%]	83.1%	[81.9%:84.2%]	5,550	86.4%
40-49	2,476	87.8%	[86.4%:89.1%]	86.1%	[84.5%:87.5%]	2,476	87.8%
15-39	-	-	-	-	-	-	-
**Carcinoma of urinary tract**							
5-14	10					10	
15-19	12					12	
5-19	37					37	
20-24	84	90.6%	[81.9%:95.4%]	91.0%	[82.2%:95.7%]	84	90.6%
25-29	203	89.6%	[84.3%:93.2%]	84.8%	[78.1%:89.7%]	203	89.6%
30-34	480	85.4%	[81.8%:88.4%]	80.9%	[76.5%:84.6%]	480	85.4%
35-39	2,855	80.0%	[78.4%:81.6%]	75.7%	[73.8%:77.5%]	2,855	80.0%
40-49	814	87.2%	[84.6%:89.4%]	83.1%	[79.9%:85.9%]	814	87.2%
15-39	10					10	
**Other invasive carcinomas**							
5-14<br>15-19	5						
5-19	12						
20-24	21						
25-29	49						
30-34	57	64.2%	[50.0%:75.3%]	61.8%	[47.2%:73.6%]	57	64.2%
35-39	149	46.6%	[37.8%:54.9%]	42.9%	[33.8%:51.7%]	149	46.6%
40-49	829	35.6%	[32.3%:39.0%]	32.1%	[28.7%:35.6%]	829	35.6%
15-39	288	53.6%	[47.4%:59.4%]	49.8%	[43.4%:56.0%]	288	53.6%
**Other Neoplasms**							
5-14	120	80.0%	[71.0%:86.4%]	77.0%	[67.3%:84.2%]	120	80.0%
15-19	26					26	
5-19	59	94.9%	[84.6%:98.5%]	95.2%	[84.8%:98.8%]	59	94.9%
20-24	62	88.1%	[76.3%:94.3%]	86.0%	[73.3%:93.1%]	62	88.1%
25-29	99	86.7%	[78.0%:92.2%]	85.3%	[75.7%:91.4%]	99	86.7%
30-34	173	85.4%	[78.9%:90.1%]	81.8%	[74.4%:87.4%]	173	85.4%
35-39	786	65.9%	[62.3%:69.3%]	63.0%	[59.1%:66.6%]	786	65.9%
40-49	415	87.3%	[83.5%:90.2%]	84.8%	[80.5%:88.2%]	415	87.3%
15-39	120	80.0%	[71.0%:86.4%]	77.0%	[67.3%:84.2%]	120	80.0%

Source: Belgian Cancer Registry. Abbreviations: CI = confidential interval
