# Supplementary material for: Cancer Burden in Adolescents and Young Adults in Belgium: Trends to Incidence Stabilisation in Recent Years with Improved Survival
Source: Cancers (Basel). 2025 May 1;17(9):1543. doi: 10.3390/cancers17091543 (PMC12071148; doi:10.3390/cancers17091543)
Supplement: Supplementary file 1 [file cancers-17-01543-s001.zip › Table S6 Prevalence.pdf]

Table S6. 5- and 10-year prevalence, by age category and by sex, Belgium, 2004-2020.

Age group	5-year prevalence N	CR	ESR 1976	ESR 2013	WSR	10-year prevalence N	CR	ESR 1976	ESR 2013	WSR
Males										
5-14 years	394	57.9	58.1	58.1	58.7	782	114.9	115.0	115.0	115.2
15-19 years	285	87.3	87.3	87.3	87.3	440	134.8	134.8	134.8	134.8
20-24 years	468	137.7	137.7	137.7	137.7	736	216.6	216.6	216.6	216.6
25-29 years	753	205.0	205.0	205.0	205.0	1,191	324.2	324.2	324.2	324.2
30-34 years	1,040	275.8	275.8	275.8	275.8	1,716	455.1	455.1	455.1	455.1
35-39 years	1,383	372.0	372.0	372.0	372.0	2,275	612.0	612.0	612.0	612.0
40-49 years	4,501	594.8	594.1	594.1	594.1	7,090	936.9	936.0	936.0	936.0
15-39 years	3,929	220.4	215.6	223.7	200.4	6,358	356.7	348.6	362.2	322.8
Females										
5-14 years	329	50.6	50.7	50.7	51.1	652	100.3	100.3	100.3	100.3
15-19 years	243	78.2	78.2	78.2	78.2	359	115.5	115.5	115.5	115.5
20-24 years	418	126.9	126.9	126.9	126.9	646	196.2	196.2	196.2	196.2
25-29 years	820	225.3	225.3	225.3	225.3	1,281	351.9	351.9	351.9	351.9
30-34 years	1,587	419.8	419.8	419.8	419.8	2,396	633.8	633.8	633.8	633.8
35-39 years	2,725	731.6	731.6	731.6	731.6	4,152	1,114.8	1,114.8	1,114.8	1,114.8
40-49 years	11,008	1,476.3	1,476.6	1,476.6	1,476.6	17,179	2,303.9	2,304.5	2,304.5	2,304.5
15-39 years	5,793	330.2	316.4	335.3	281.9	8,834	503.5	482.4	511.2	430.2
**Hodgkin Lymphoma**										
5-14 years	40	3.0	3.0	3.0	2.9	48	3.6	3.6	3.6	3.5
15-19 years	111	17.4	17.4	17.4	17.4	140	22.0	22.0	22.0	22.0
20-24 years	186	27.8	27.8	27.8	27.8	278	41.6	41.6	41.6	41.6
25-29 years	178	24.3	24.3	24.3	24.3	366	50.0	50.0	50.0	50.0
30-34 years	163	21.6	21.6	21.6	21.6	340	45.0	45.0	45.0	45.0
35-39 years	157	21.1	21.1	21.1	21.1	298	40.0	40.0	40.0	40.0
40-49 years	189	12.6	12.6	12.6	12.6	439	29.2	29.2	29.2	29.2
15-39 years	795	22.5	22.4	22.5	22.4	1,422	40.2	39.7	40.1	38.9
**Mature B-cell neoplasms**										
5-14 years	43	3.2	3.2	3.2	3.3	82	6.2	6.1	6.1	6.0
15-19 years	37	5.8	5.8	5.8	5.8	59	9.3	9.3	9.3	9.3
20-24 years	47	7.0	7.0	7.0	7.0	72	10.8	10.8	10.8	10.8
25-29 years	47	6.4	6.4	6.4	6.4	97	13.3	13.3	13.3	13.3
30-34 years	104	13.8	13.8	13.8	13.8	170	22.5	22.5	22.5	22.5
35-39 years	168	22.6	22.6	22.6	22.6	262	35.2	35.2	35.2	35.2
40-49 years	681	45.3	45.3	45.3	45.3	1,050	69.9	69.8	69.8	69.8
15-39 years	403	11.4	11.1	11.6	10.2	660	18.7	18.2	19.0	16.8
**Mature T- and NK-cell neoplasms**										
5-14 years	10	0.8	0.8	0.8	0.8	14	1.1	1.0	1.0	1.0
15-19 years	13	2.0	2.0	2.0	2.0	16	2.5	2.5	2.5	2.5
20-24 years	11	1.6	1.6	1.6	1.6	22	3.3	3.3	3.3	3.3
25-29 years	23	3.1	3.1	3.1	3.1	36	4.9	4.9	4.9	4.9
30-34 years	29	3.8	3.8	3.8	3.8	48	6.4	6.4	6.4	6.4
35-39 years	38	5.1	5.1	5.1	5.1	61	8.2	8.2	8.2	8.2
40-49 years	104	6.9	6.9	6.9	6.9	167	11.1	11.1	11.1	11.1
15-39 years	114	3.2	3.2	3.2	3.0	183	5.2	5.1	5.2	4.7
**Other lymphoid neoplasms**										
5-14 years	1	0.1	0.1	0.1	0.1	1	0.1	0.1	0.1	0.1

15-19 years	-	-	-	-	-	-	-	-	-	-
20-24 years	3	0.4	0.4	0.4	0.4	6	0.9	0.9	0.9	0.9
25-29 years	8	1.1	1.1	1.1	1.1	12	1.6	1.6	1.6	1.6
30-34 years	6	0.8	0.8	0.8	0.8	13	1.7	1.7	1.7	1.7
35-39 years	5	0.7	0.7	0.7	0.7	8	1.1	1.1	1.1	1.1
40-49 years	14	0.9	0.9	0.9	0.9	24	1.6	1.6	1.6	1.6
15-39 years	22	0.6	0.6	0.6	0.6	39	1.1	1.1	1.1	1.0
**Precursor hematopoietic neoplasms**										
5-14 years	270	20.3	20.4	20.4	20.8	528	39.7	39.7	39.7	39.8
15-19 years	77	12.1	12.1	12.1	12.1	138	21.7	21.7	21.7	21.7
20-24 years	50	7.5	7.5	7.5	7.5	115	17.2	17.2	17.2	17.2
25-29 years	66	9.0	9.0	9.0	9.0	121	16.5	16.5	16.5	16.5
30-34 years	46	6.1	6.1	6.1	6.1	84	11.1	11.1	11.1	11.1
35-39 years	56	7.5	7.5	7.5	7.5	96	12.9	12.9	12.9	12.9
40-49 years	141	9.4	9.4	9.4	9.4	229	15.2	15.2	15.2	15.2
15-39 years	295	8.3	8.4	8.3	8.7	554	15.7	15.9	15.6	16.5
**Chronic myeloid neoplasms**										
5-14 years	30	2.3	2.2	2.2	2.2	61	4.6	4.6	4.6	4.5
15-19 years	13	2.0	2.0	2.0	2.0	26	4.1	4.1	4.1	4.1
20-24 years	31	4.6	4.6	4.6	4.6	55	8.2	8.2	8.2	8.2
25-29 years	56	7.7	7.7	7.7	7.7	79	10.8	10.8	10.8	10.8
30-34 years	78	10.3	10.3	10.3	10.3	122	16.2	16.2	16.2	16.2
35-39 years	127	17.1	17.1	17.1	17.1	207	27.8	27.8	27.8	27.8
40-49 years	402	26.8	26.7	26.7	26.7	637	42.4	42.4	42.4	42.4
15-39 years	305	8.6	8.3	8.8	7.6	489	13.8	13.4	14.1	12.2
**Histiocytic and dendritic cell neoplasms (HDCN)**										
5-14 years	39	2.9	2.9	2.9	2.9	88	6.6	6.6	6.6	6.7
15-19 years	4	0.6	0.6	0.6	0.6	15	2.4	2.4	2.4	2.4
20-24 years	12	1.8	1.8	1.8	1.8	22	3.3	3.3	3.3	3.3
25-29 years	7	1.0	1.0	1.0	1.0	18	2.5	2.5	2.5	2.5
30-34 years	8	1.1	1.1	1.1	1.1	17	2.3	2.3	2.3	2.3
35-39 years	8	1.1	1.1	1.1	1.1	15	2.0	2.0	2.0	2.0
40-49 years	21	1.4	1.4	1.4	1.4	39	2.6	2.6	2.6	2.6
15-39 years	39	1.1	1.1	1.1	1.1	87	2.5	2.5	2.5	2.5
**CNS**										
5-14 years	95	7.1	7.2	7.2	7.2	170	12.8	12.8	12.8	12.8
15-19 years	53	8.3	8.3	8.3	8.3	98	15.4	15.4	15.4	15.4
20-24 years	52	7.8	7.8	7.8	7.8	88	13.2	13.2	13.2	13.2
25-29 years	75	10.3	10.3	10.3	10.3	124	17.0	17.0	17.0	17.0
30-34 years	99	13.1	13.1	13.1	13.1	154	20.4	20.4	20.4	20.4
35-39 years	127	17.1	17.1	17.1	17.1	212	28.5	28.5	28.5	28.5
40-49 years	323	21.5	21.5	21.5	21.5	488	32.5	32.5	32.5	32.5
15-39 years	406	11.5	11.3	11.6	10.8	676	19.1	18.9	19.3	18.2
**Sarcoma**										
5-14 years	77	5.8	5.8	5.8	5.8	129	9.7	9.7	9.7	9.7
15-19 years	83	13.0	13.0	13.0	13.0	130	20.4	20.4	20.4	20.4
20-24 years	67	10.0	10.0	10.0	10.0	137	20.5	20.5	20.5	20.5
25-29 years	77	10.5	10.5	10.5	10.5	154	21.1	21.1	21.1	21.1
30-34 years	121	16.0	16.0	16.0	16.0	200	26.5	26.5	26.5	26.5
35-39 years	149	20.0	20.0	20.0	20.0	255	34.3	34.3	34.3	34.3
40-49 years	469	31.2	31.2	31.2	31.2	778	51.8	51.8	51.8	51.8
15-39 years	497	14.1	13.9	14.2	13.5	876	24.8	24.5	24.9	23.8

**Skin melanoma**										
5-14 years	6	0.5	0.4	0.4	0.4	8	0.6	0.6	0.6	0.6
15-19 years	16	2.5	2.5	2.5	2.5	20	3.1	3.1	3.1	3.1
20-24 years	102	15.2	15.2	15.2	15.2	125	18.7	18.7	18.7	18.7
25-29 years	255	34.9	34.9	34.9	34.9	349	47.7	47.7	47.7	47.7
30-34 years	445	58.9	58.9	58.9	58.9	725	96.0	96.0	96.0	96.0
35-39 years	664	89.2	89.2	89.2	89.2	1,034	138.9	138.9	138.9	138.9
40-49 years	2,136	142.2	142.1	142.1	142.1	3,529	234.9	234.8	234.8	234.8
15-39 years	1,482	41.9	40.2	42.6	35.5	2,253	63.7	60.9	64.9	53.2
**All gonadal and related cancers**										
5-14 years	23	1.7	1.7	1.7	1.7	53	4.0	4.0	4.0	4.0
15-19 years	41	6.4	6.4	6.4	6.4	49	7.7	7.7	7.7	7.7
20-24 years	156	23.3	23.3	23.3	23.3	211	31.5	31.5	31.5	31.5
25-29 years	316	43.2	43.2	43.2	43.2	447	61.1	61.1	61.1	61.1
30-34 years	428	56.7	56.7	56.7	56.7	704	93.2	93.2	93.2	93.2
35-39 years	425	57.1	57.1	57.1	57.1	814	109.4	109.4	109.4	109.4
40-49 years	655	43.6	43.6	43.6	43.6	1,314	87.5	87.5	87.5	87.5
15-39 years	1,366	38.6	37.4	38.8	34.4	2,225	62.9	60.6	63.5	54.8
**Testis**										
5-14 years	2	0.2	0.2	0.2	0.2	11	0.8	0.8	0.8	0.8
15-19 years	16	2.5	2.5	2.5	2.5	17	2.7	2.7	2.7	2.7
20-24 years	134	20.0	20.0	20.0	20.0	162	24.2	24.2	24.2	24.2
25-29 years	275	37.6	37.6	37.6	37.6	373	51.0	51.0	51.0	51.0
30-34 years	377	49.9	49.9	49.9	49.9	607	80.4	80.4	80.4	80.4
35-39 years	366	49.2	49.2	49.2	49.2	710	95.4	95.4	95.4	95.4
40-49 years	468	31.2	31.2	31.2	31.2	1,018	67.8	67.8	67.8	67.8
15-39 years	1,168	33.0	31.9	33.2	29.1	1,869	52.8	50.7	53.4	45.4
**Ovary**										
5-14 years	8	0.6	0.6	0.6	0.6	10	0.8	0.7	0.7	0.7
15-19 years	11	1.7	1.7	1.7	1.7	14	2.2	2.2	2.2	2.2
20-24 years	12	1.8	1.8	1.8	1.8	30	4.5	4.5	4.5	4.5
25-29 years	28	3.8	3.8	3.8	3.8	48	6.6	6.6	6.6	6.6
30-34 years	24	3.2	3.2	3.2	3.2	50	6.6	6.6	6.6	6.6
35-39 years	40	5.4	5.4	5.4	5.4	69	9.3	9.3	9.3	9.3
40-49 years	164	10.9	10.9	10.9	10.9	248	16.5	16.5	16.5	16.5
15-39 years	115	3.3	3.2	3.3	3.0	211	6.0	5.8	6.0	5.5
**Germ cell and trophoblastic – CNS**										
5-14 years	8	0.6	0.6	0.6	0.6	15	1.1	1.1	1.1	1.1
15-19 years	9	1.4	1.4	1.4	1.4	13	2.0	2.0	2.0	2.0
20-24 years	6	0.9	0.9	0.9	0.9	15	2.2	2.2	2.2	2.2
25-29 years	3	0.4	0.4	0.4	0.4	10	1.4	1.4	1.4	1.4
30-34 years	4	0.5	0.5	0.5	0.5	9	1.2	1.2	1.2	1.2
35-39 years	4	0.5	0.5	0.5	0.5	7	0.9	0.9	0.9	0.9
40-49 years	5	0.3	0.3	0.3	0.3	8	0.5	0.5	0.5	0.5
15-39 years	26	0.7	0.8	0.7	0.8	54	1.5	1.6	1.5	1.6
**Germ cell and trophoblastic excluding CNS, ovary, testis**										
5-14 years	5	0.4	0.4	0.4	0.4	17	1.3	1.3	1.3	1.4
15-19 years	5	0.8	0.8	0.8	0.8	5	0.8	0.8	0.8	0.8
20-24 years	4	0.6	0.6	0.6	0.6	4	0.6	0.6	0.6	0.6
25-29 years	10	1.4	1.4	1.4	1.4	15	2.1	2.1	2.1	2.1
30-34 years	23	3.0	3.0	3.0	3.0	38	5.0	5.0	5.0	5.0
35-39 years	15	2.0	2.0	2.0	2.0	28	3.8	3.8	3.8	3.8

40-49 years	16	1.1	1.1	1.1	1.1	38	2.5	2.5	2.5	2.5
15-39 years	57	1.6	1.6	1.6	1.4	90	2.5	2.4	2.6	2.2
**All carcinomas**										
5-14 years	57	4.3	4.2	4.2	4.1	66	5.0	4.9	4.9	4.7
15-19 years	117	18.4	18.4	18.4	18.4	157	24.6	24.6	24.6	24.6
20-24 years	265	39.6	39.6	39.6	39.6	395	59.0	59.0	59.0	59.0
25-29 years	752	102.8	102.8	102.8	102.8	1,060	144.9	144.9	144.9	144.9
30-34 years	1,846	244.5	244.5	244.5	244.5	2,549	337.6	337.6	337.6	337.6
35-39 years	3,613	485.5	485.5	485.5	485.5	5,238	703.9	703.9	703.9	703.9
40-49 years	18,436	1,227.1	1,226.3	1,226.3	1,226.3	27,196	1,810.2	1,809.0	1,809.0	1,809.0
15-39 years	6,593	186.4	178.2	191.7	153.6	9,399	265.7	254.0	273.6	219.0
**Thyroid carcinoma**										
5-14 years	9	0.7	0.7	0.7	0.6	9	0.7	0.7	0.7	0.6
15-19 years	30	4.7	4.7	4.7	4.7	40	6.3	6.3	6.3	6.3
20-24 years	70	10.5	10.5	10.5	10.5	102	15.2	15.2	15.2	15.2
25-29 years	157	21.5	21.5	21.5	21.5	247	33.8	33.8	33.8	33.8
30-34 years	236	31.3	31.3	31.3	31.3	406	53.8	53.8	53.8	53.8
35-39 years	359	48.2	48.2	48.2	48.2	613	82.4	82.4	82.4	82.4
40-49 years	875	58.2	58.2	58.2	58.2	1,671	111.2	111.2	111.2	111.2
15-39 years	852	24.1	23.2	24.5	20.9	1,408	39.8	38.3	40.5	34.2
**Other carcinoma of head and neck**										
5-14 years	3	0.2	0.2	0.2	0.2	3	0.2	0.2	0.2	0.2
15-19 years	8	1.3	1.3	1.3	1.3	13	2.0	2.0	2.0	2.0
20-24 years	13	1.9	1.9	1.9	1.9	18	2.7	2.7	2.7	2.7
25-29 years	22	3.0	3.0	3.0	3.0	35	4.8	4.8	4.8	4.8
30-34 years	50	6.6	6.6	6.6	6.6	69	9.1	9.1	9.1	9.1
35-39 years	72	9.7	9.7	9.7	9.7	113	15.2	15.2	15.2	15.2
40-49 years	394	26.2	26.2	26.2	26.2	572	38.1	38.0	38.0	38.0
15-39 years	165	4.7	4.5	4.8	4.0	248	7.0	6.8	7.2	6.1
**Carcinoma of gastrointestinal tract**										
5-14 years	23	1.7	1.7	1.7	1.6	26	2.0	1.9	1.9	1.8
15-19 years	53	8.3	8.3	8.3	8.3	67	10.5	10.5	10.5	10.5
20-24 years	73	10.9	10.9	10.9	10.9	128	19.1	19.1	19.1	19.1
25-29 years	108	14.8	14.8	14.8	14.8	198	27.1	27.1	27.1	27.1
30-34 years	222	29.4	29.4	29.4	29.4	326	43.2	43.2	43.2	43.2
35-39 years	352	47.3	47.3	47.3	47.3	486	65.3	65.3	65.3	65.3
40-49 years	1,445	96.2	96.1	96.1	96.1	2,077	138.2	138.2	138.2	138.2
15-39 years	808	22.8	22.1	23.3	20.0	1,205	34.1	33.0	34.6	30.1
**Colorectal carcinomas**										
5-14 years	19	1.4	1.4	1.4	1.3	20	1.5	1.5	1.5	1.4
15-19 years	47	7.4	7.4	7.4	7.4	60	9.4	9.4	9.4	9.4
20-24 years	60	9.0	9.0	9.0	9.0	112	16.7	16.7	16.7	16.7
25-29 years	81	11.1	11.1	11.1	11.1	157	21.5	21.5	21.5	21.5
30-34 years	156	20.7	20.7	20.7	20.7	246	32.6	32.6	32.6	32.6
35-39 years	255	34.3	34.3	34.3	34.3	353	47.4	47.4	47.4	47.4
40-49 years	917	61.0	61.0	61.0	61.0	1,371	91.3	91.2	91.2	91.2
15-39 years	599	16.9	16.5	17.3	15.0	928	26.2	25.5	26.6	23.5
**Rest of the carcinoma of the gastrointestinal tract**										
5-14 years	4	0.3	0.3	0.3	0.3	6	0.5	0.4	0.4	0.4
15-19 years	6	0.9	0.9	0.9	0.9	7	1.1	1.1	1.1	1.1
20-24 years	13	1.9	1.9	1.9	1.9	16	2.4	2.4	2.4	2.4
25-29 years	27	3.7	3.7	3.7	3.7	41	5.6	5.6	5.6	5.6

30-34 years	66	8.7	8.7	8.7	8.7	80	10.6	10.6	10.6	10.6
35-39 years	97	13.0	13.0	13.0	13.0	133	17.9	17.9	17.9	17.9
40-49 years	530	35.3	35.3	35.3	35.3	709	47.2	47.2	47.2	47.2
15-39 years	209	5.9	5.7	6.0	5.0	277	7.8	7.5	8.0	6.6
**Carcinoma of lung, bronchus and trachea**										
5-14 years	2	0.2	0.1	0.1	0.1	3	0.2	0.2	0.2	0.2
15-19 years	3	0.5	0.5	0.5	0.5	5	0.8	0.8	0.8	0.8
20-24 years	8	1.2	1.2	1.2	1.2	13	1.9	1.9	1.9	1.9
25-29 years	10	1.4	1.4	1.4	1.4	18	2.5	2.5	2.5	2.5
30-34 years	25	3.3	3.3	3.3	3.3	35	4.6	4.6	4.6	4.6
35-39 years	67	9.0	9.0	9.0	9.0	87	11.7	11.7	11.7	11.7
40-49 years	428	28.5	28.5	28.5	28.5	512	34.1	34.0	34.0	34.0
15-39 years	113	3.2	3.1	3.3	2.7	158	4.5	4.3	4.6	3.8
**Carcinoma of skin**										
5-14 years	16	1.2	1.2	1.2	1.2	20	1.5	1.5	1.5	1.4
15-19 years	21	3.3	3.3	3.3	3.3	30	4.7	4.7	4.7	4.7
20-24 years	79	11.8	11.8	11.8	11.8	107	16.0	16.0	16.0	16.0
25-29 years	278	38.0	38.0	38.0	38.0	365	49.9	49.9	49.9	49.9
30-34 years	706	93.5	93.5	93.5	93.5	955	126.5	126.5	126.5	126.5
35-39 years	1,414	190.0	190.0	190.0	190.0	2,033	273.2	273.2	273.2	273.2
40-49 years	8,106	539.5	539.2	539.2	539.2	11,677	777.2	776.7	776.7	776.7
15-39 years	2,498	70.6	67.3	72.7	57.5	3,490	98.7	94.1	101.8	80.2
**Carcinoma of breast**										
5-14 years	-	-	-	-	-	-	-	-	-	-
15-19 years	-	-	-	-	-	-	-	-	-	-
20-24 years	9	1.3	1.3	1.3	1.3	9	1.3	1.3	1.3	1.3
25-29 years	129	17.6	17.6	17.6	17.6	141	19.3	19.3	19.3	19.3
30-34 years	450	59.6	59.6	59.6	59.6	527	69.8	69.8	69.8	69.8
35-39 years	1,011	135.9	135.9	135.9	135.9	1,393	187.2	187.2	187.2	187.2
40-49 years	5,704	379.7	379.4	379.4	379.4	8,476	564.2	563.8	563.8	563.8
15-39 years	1,599	45.2	42.9	46.8	35.8	2,070	58.5	55.5	60.9	46.1
**Carcinoma of genital sites excluding ovary and testis**										
5-14 years	-	-	-	-	-	-	-	-	-	-
15-19 years	-	-	-	-	-	-	-	-	-	-
20-24 years	3	0.4	0.4	0.4	0.4	3	0.4	0.4	0.4	0.4
25-29 years	28	3.8	3.8	3.8	3.8	30	4.1	4.1	4.1	4.1
30-34 years	131	17.3	17.3	17.3	17.3	191	25.3	25.3	25.3	25.3
35-39 years	258	34.7	34.7	34.7	34.7	409	55.0	55.0	55.0	55.0
40-49 years	1,025	68.2	68.2	68.2	68.2	1,677	111.6	111.6	111.6	111.6
15-39 years	420	11.9	11.3	12.3	9.4	633	17.9	17.0	18.6	14.0
**Carcinoma of urinary tract**										
5-14 years	3	0.2	0.2	0.2	0.2	3	0.2	0.2	0.2	0.2
15-19 years	2	0.3	0.3	0.3	0.3	2	0.3	0.3	0.3	0.3
20-24 years	9	1.3	1.3	1.3	1.3	12	1.8	1.8	1.8	1.8
25-29 years	17	2.3	2.3	2.3	2.3	21	2.9	2.9	2.9	2.9
30-34 years	27	3.6	3.6	3.6	3.6	40	5.3	5.3	5.3	5.3
35-39 years	73	9.8	9.8	9.8	9.8	112	15.1	15.1	15.1	15.1
40-49 years	554	36.9	36.8	36.8	36.8	819	54.5	54.5	54.5	54.5
15-39 years	128	3.6	3.5	3.7	3.0	187	5.3	5.1	5.5	4.4
**Other invasive carcinomas**										
5-14 years	1	0.1	0.1	0.1	0.1	2	0.2	0.1	0.1	0.1
15-19 years	-	-	-	-	-	-	-	-	-	-

20-24 years	3	0.4	0.4	0.4	0.4	5	0.7	0.7	0.7	0.7
25-29 years	6	0.8	0.8	0.8	0.8	8	1.1	1.1	1.1	1.1
30-34 years	10	1.3	1.3	1.3	1.3	19	2.5	2.5	2.5	2.5
35-39 years	27	3.6	3.6	3.6	3.6	31	4.2	4.2	4.2	4.2
40-49 years	98	6.5	6.5	6.5	6.5	131	8.7	8.7	8.7	8.7
15-39 years	46	1.3	1.2	1.3	1.1	63	1.8	1.7	1.8	1.5
**Other neoplasms**										
5-14 years	100	7.5	7.6	7.6	7.9	292	21.9	22.1	22.1	22.5
15-19 years	5	0.8	0.8	0.8	0.8	14	2.2	2.2	2.2	2.2
20-24 years	8	1.2	1.2	1.2	1.2	11	1.6	1.6	1.6	1.6
25-29 years	18	2.5	2.5	2.5	2.5	32	4.4	4.4	4.4	4.4
30-34 years	22	2.9	2.9	2.9	2.9	33	4.4	4.4	4.4	4.4
35-39 years	43	5.8	5.8	5.8	5.8	72	9.7	9.7	9.7	9.7
40-49 years	136	9.1	9.0	9.0	9.0	215	14.3	14.3	14.3	14.3
15-39 years	96	2.7	2.6	2.8	2.4	162	4.6	4.5	4.7	4.1

Source: Belgian Cancer Registry. Abbreviations: N = number of cases, CR = crude rate (number per 100.000), WSR = world standardized rate, ESR = European standardized rate (number per 100.000), CI = confidential interval
