# Supplementary material for: Cancer Burden in Adolescents and Young Adults in Belgium: Trends to Incidence Stabilisation in Recent Years with Improved Survival
Source: Cancers (Basel). 2025 May 1;17(9):1543. doi: 10.3390/cancers17091543 (PMC12071148; doi:10.3390/cancers17091543)
Supplement: Supplementary file 1 [file cancers-17-01543-s001.zip › Table S7 International comparison.pdf]

Table S7. Comparison of Belgian incidence and mortality rates with international data.

Cancer types	Incidence											
	Belgium			Netherlands			France			Germany		
	ASR 2019	APC	AAPC 2004-2020	ASR 2019	APC	AAPC 2004-2020	ASR 2019	APC	AAPC 2004-2020	ASR 2019	APC	AAPC 2004-2020
**All cancers**	78,0 [75,0;80,9]	2004-2015 0.4* [0.3;1.0]<br>2015-2020 -0.4 [-1.7;0.1]	0.2* [0,0;0,4]	75,1 [72,8;77,4]	2004-2008 0.4* [0,1;1,4]<br>2008-2020 0.1 [-0,5;0,1]	0.1* [0,0;0,2]	78,1 [76,8;79,4]	-	0,3* [0,1;0,4]	73,1 [72,0;74,1]	2004-2008 0.3* [0,1;0,8]<br>2008-2020 0.0 [-0,1;0,0]	0.1* [0,0;0,1]
Male	61,1 [57,4;64,8]	-	0.3* [0,1;0,4]	61,1 [58,2;64,1]	2004-2011 0.4* [0,2;1,2]<br>2011-2020 -0.0 [-0,4;0,1]	0.1* [0,1;0,3]	61,2 [59,6;62,8]	2004-2007 -0.1 [-0,7;0,4]<br>2007-2010 0.9* [0,1;1,2]<br>2010-2020 0.0 [-0,1;0,1]	0,1* [0,1;0,2]	58,8 [57,4;60,1]	-	0.0 [-0,0;0,0]
Female	94,9 [90,3;99,5]	-	0.1* [0,0;0,3]	89,4 [85,8;93,0]	-	0.1* [0,0;0,2]	95,0 [93,0;97,0]	2004-2009 0.5* [0,3;1,1]<br>2009-2018 0.0 [-0,4;0,1]<br>2018-2020 1.2* [0,4;1,7]	0,3* [0,3;0,4]	88,3 [86,6;90,0]	2004-2008 0.4* [0,2;0,8]<br>2008-2020 0.1* [0,0;0,1]	0.1* [0,1;0,2]
**Hodgkin lymphoma**	5,3 [4,5;6,1]	-	1.1* [0,7;1,6]	4,0 [3,5;4,6]	-	0.5 [-0,3;1,5]	5.4 [5,0;5,7] **	-	1,2* [0,8;1,6] **	4,3 [4,1;4,6]	-	0.9* [0,4;1,3]
**Central nervous system**	3,4 [2,8;4,1]	-	-0.3 [-1,5;0,8]	2,8 [2,3;3,2]	-	-	2,8 [2,6;3,1]	2004-2009 2.7* [1,3;5,2]<br>2009-2020 -1.2* [-1,8;-0,8]	0,0 [-0,4;0,4]	3,1 [2,9;3,3]	-	-0.2 [-0,6;0,2]
**Skin melanoma**	11,5 [10,4;12,6]	2004-2010 1.99* [0.8;9.0]<br>2010-2020 -0.02 [-3.1;0.5]	0.7* [0,2;1,3]	10,1 [9,2;10,9]	2004-2009 0.8 [-0,3;4,5]<br>2009-2020 -0.9* [-3,1;-0,5]	-0.4 [-0,8;0,0]	7,6 [7,2;8,0]	2004-2010 1.5* [0,9;2,9]<br>2010-2020 -0.6* [-1,1;-0,3]	0,2 [-0,0;0,4]	8,0 [7,6;8,3]	2004-2011 2.0* [1,4;3,0]<br>2011-2020 -1.7* [-2,3;-1,2]	-0.1 [-0,3;0,2]
**Testicular cancer**	7,2 [6,3;8,1]	-	1.1* [0,7;1,6]	10,6 [9,8;11,5]	2004-2014 1.4* [1,1;4,3]<br>2014-2020 0.2 [-2,2;0,8]	0.9* [0,6;1,3]	10.7 [10,2;11,1] **	2004-2006 -1.4 [-6,1;4,8]<br>2006-2009 5.1 [-1,7;8,2]<br>2009-2018 1.0 [-2,5;4,3]	1,5* [1,1;2,4] **	9,8 [9,4;10,2]	-	0.0 [-0,1;0,2]
**Thyroid cancer**	5,4 [4,7;6,2]	2004-2012 4,0* [2,6;6,7]<br>2012-2020 -1,3* [-3,2;-0,2]	1.3* [0,7;2,1]	3,2 [2,8;3,7]	2004-2014 4.2* [3,0;9,8]<br>2014-2020 -0.7 [-7,0;1,5]	2.3* [1,3;3,8]	8.8 [8,4;9,3] **	2004-2013 1.7* [1,3;2,3]<br>2013-2018 -2.0* [-3,4;-1,1]	0,4* [0,0;0,7]	6,1 [5,8;6,4]	2004-2009 4.8* [3,3;7,3]<br>2009-2020 0.3 [-0,1;0,7]	1.7* [1,4;2,1]
**Colorectal cancer**	4,8 [4,0;5,5]	2004-2013 3,4* [2,1;11,1]<br>2013-2020 -0,4 [-5,0;1,2]	1.7* [0,9;3,0]	2,5 [2,1;2,9]	-	0.2 [-0,8;1,3]	4,2 [4,0;4,5]	-	2,8* [2,4;3,3]	4,1 [3,9;4,4]	2004-2009 -2.4* [-4,2;-1,3]<br>2009-2020 2.0* [1,7;2,5]	0.6* [0,4;0,9]
**Breast cancer**	15,4 [14,0;16,7]	-	0.3* [0,1;0,5]	14,6 [13,6;15,7]	-	0.3* [0,1;0,4]	15,9 [15,3;16,5]	-	0,5* [0,3;0,7]	13,2 [12,8;13,7]	2004-2008 0.8* [0,2;2,5]<br>2008-2014 -0.4* [-1,6;-0,1]<br>2014-2020 0.9* [0,4;2,3]	0.3* [0,2;0,5]

Source: 1) Belgium: Belgian Cancer Registry, 2) Netherlands: cancer data https://nkr-cijfers.iknl.nl/, population data https://www.cbs.nl/, 3) France: https://www.e-cancer.fr/, 4) Germany: cancer data https://www.krebsdaten.de/, population data https://www.destatis.de/. **ASR** = age-standardized rate (number per 100,000), calculated from the revised European Standard Population. * Indicates that the AAPC is significantly different from zero at alpha = 0.05 level. **Data available until 2018.

Cancer types	Mortality								
	Belgium		Netherlands		France		Germany		
	ASR 2019	AAPC 2004-2019	ASR 2019	AAPC 2004-2019	ASR 2015	AAPC 2004-2015	ASR 2019	APC	AAPC 2004-20219
**All cancers**	6,7 [5,8;7,5]	-1,16* [-1,72;-0,69]	7,4 [6,7;8,1]	-1,4* [-1,6;-1,2]	9,4 [8,9;9,8]	-0,76* [-1,18;-0,38]	8,0 [7,6;8,3]	2004-2015 -1,1* [-1,5;-0,9]<br>2015-2019 0,7 [-0,3;2,8]	-0,6* [-0,8;-0,4]
Male	6,3 [5,1;7,5]	-0,88* [-1,72;-0,17]	7,5 [6,5;8,5]	-1,2* [-1,7;-0,9]	9,7 [9,1;10,4]	-0,17 [-0,45;0,11]	6,9 [6,4;7,4]	-	-1,0* [-1,2;-0,9]
Female	7,0 [5,8;8,3]	-1,13* [-1,59;-0,74]	7,3 [6,3;8,3]	-1,4* [-1,7;-1,2]	9,0 [8,4;9,6]	-0,76* [-1,29;-0,27]	9,1 [8,6;9,7]	2004-2015 -1,0* [-1,6;-0,7]<br>2015-2019 1,5* [0,0;4,4]	-0,3* [-0,6;-0,1]
**Hodgkin lymphoma**	0,9 [0,6;1,2]	-	1,0 [0,7;1,2]	-	-	-	-	-	-
**Central nervous system**	0,9 [0,6;1,2]	-	1,2 [0,9;1,5]	-4,8* [-8,2;-2,7]	1,1 [1,0;1,3]	-	1,0 [0,9;1,2]	-	-
**Skin melanoma**	0,2 [0,0;0,3]	-	0,4 [0,2;0,6]	-	0,4 [0,3;0,5]	-	0,3 [0,2;0,3]	-	-
**Testicular cancer**	-	-	0,1 [0,0;0,2]	-	0,3 [0,2;0,5]	-	0,1 [0,1;0,2]	-	-
**Thyroid cancer**	0,1 [0,0;0,1]	-	0,0 [0,0;0,1]	-	0,0 [0,0;0,1]	-	0,0 [0,0;0,0]	-	-
**Colorectal cancer**	0,6 [0,4;0,9]	-	0,4 [0,2;0,5]	-	0,7 [0,6;0,8]	-	0,6 [0,5;0,7]	-	-
**Breast cancer**	0,6 [0,3;0,9]	-	0,9 [0,6;1,1]	-	1,2 [1,1;1,4]	-	1,3 [1,1;1,4]	-	-

Source: 1) Belgium: Belgian Cancer Registry, 2) Netherlands: cancer data https://nkr-cijfers.iknl.nl/, population data https://www.cbs.nl/, 3) France: https://www.e-cancer.fr/, 4) Germany: cancer data https://www.krebsdaten.de/, population data https://www.destatis.de/. **ASR** = age-standardized rate (number per 100,000), calculated from the revised European Standard Population. * Indicates that the AAPC is significantly different from zero at alpha = 0.05 level.
